# NEAT: a framework for building fully automated NGS pipelines and analyses

**DOI:** 10.1186/s12859-016-0902-3

**Published:** 2016-02-01

**Authors:** Patrick Schorderet

**Affiliations:** Department of Molecular Biology, Massachusetts General Hospital, Boston, MA 02114 USA; Department of Genetics, Harvard Medical School, Boston, MA 02115 USA

**Keywords:** Bioinformatics, Genomics, High throughput sequencing, RNAseq, ChIPseq, NGS pipelines

## Abstract

**Background:**

The analysis of next generation sequencing (NGS) has become a standard task for many laboratories in the life sciences. Though there exists several tools to support users in the manipulation of such datasets on various levels, few are built on the basis of vertical integration. Here, we present the *NE*xt generation *A*nalysis *T*oolbox (NEAT) that allows non-expert users including wet-lab scientists to comprehensively build, run and analyze NGS data through double-clickable executables without the need of any programming experience.

**Results:**

In comparison to many publicly available tools including Galaxy, NEAT provides three main advantages: (1) Through the development of double-clickable executables, NEAT is efficient (completes within <24 hours), easy to implement and intuitive; (2) Storage space, maximum number of job submissions, wall time and cluster-specific parameters can be customized as NEAT is run on the institution’s cluster; (3) NEAT allows users to visualize and summarize NGS data rapidly and efficiently using various built-in exploratory data analysis tools including metagenomic and differentially expressed gene analysis.

To simplify the control of the workflow, NEAT projects are built around a unique and centralized file containing sample names, replicates, conditions, antibodies, alignment-, filtering- and peak calling parameters as well as cluster-specific paths and settings. Moreover, the small-sized files produced by NEAT allow users to easily manipulate, consolidate and share datasets from different users and institutions.

**Conclusions:**

NEAT provides biologists and bioinformaticians with a robust, efficient and comprehensive tool for the analysis of massive NGS datasets. Frameworks such as NEAT not only allow novice users to overcome the increasing number of technical hurdles due to the complexity of manipulating large datasets, but provide more advance users with tools that ensure high reproducibility standards in the NGS era. NEAT is publically available at https://github.com/pschorderet/NEAT.

**Electronic supplementary material:**

The online version of this article (doi:10.1186/s12859-016-0902-3) contains supplementary material, which is available to authorized users.

## Background

Massively parallel / next generation sequencing (NGS) has become a central tool for many projects related to the life sciences, including fields such as molecular biology, evolutionary biology, metagenomics and oncology. These novel technologies have brought tremendous depth to our understanding of epigenetics and are becoming widely used in many experimental setups. Recent improvements in sequencing technologies have made it commonplace to obtain 20 to 40 gigabits of data from a single experiment [1] while the cost per mega base has dropped by half nearly every six months since 2008 [2, 3]. The explosion of NGS data in the life sciences has lead to surpass the petabase barrier [4]. In addition to the massive amount of data generated in the genomics era, the empiric observation that NGS analysis constitutes one of the major bottlenecks in modern genomics projects has brought new challenges including the urgent need to create efficient and reproducible analysis pipelines accessible to both biologists and bioinformaticians.

Biologists have embraced NGS technologies with great enthusiasm, mainly because of the opportunities and promises they provide. However, although NGS allows rapid assessment of genome wide changes, paradoxically, the computational power and complexity required for its analysis has significantly hindered the overall turnaround time for wet-lab scientists, many of whom rely on overwhelmed bioinformatics core facilities. A common effort has thus been established to support the post-genomic era including the development of important interfaces such as genome browsers (UCSC [5–7], Ensembl [8]), annotation databases (ENCODE [9], modENCODE [10]) and tools to manipulate big data files (BEDTools [11], SAMtools [12]). Moreover, many scientists have contributed to the development of Galaxy, an open source, web-based platform that provides various tools for NGS data analysis [13, 14]. Finally, the R community is providing increased support to the field of bioinformatics by developing and providing a plethora of open source packages as part of the Bioconductor consortium [15].

The development of publically available tools has undoubtedly facilitated the analysis of NGS data. However, several loopholes still remain. For example, irrespective of how user-friendly these tools might be, they are often daunting for scientists that have little to no programming experience. This particular segment is often brought to the dilemma of choosing between investing the effort to learn the computational skills necessary to analyze their own data or waiting for it to be analyzed by computational cores. Empirically, the majority of the decisions converge to the later. In the meantime, scientists still heavily rely on the ability to visualize their data to steer their projects. We thus feel that the community would strongly benefit from easy-to-use tools that do not require programming skills. The reason such applications have never been implemented likely stems from the disparity of each individual project and the need to apply specific parameters to each of them on a case-by-case scenario. Nevertheless, there is a strong demand for tools to rapidly assess whether the technical aspect of an experiment succeeded (antibody specificity, conditions, sequencing depth, etc), even though the tradeoff of using default parameters might well introduces some bias and imperfections in the analysis.

Another loophole in NGS analysis is seen with more advanced users. Indeed, many computational biologists, who strongly depend on automation for the majority of their work, continue to manually manipulate files (renaming, filing, copying, etc). This apparent dichotomy can be explained by the lack of tools to support vertical integration of NGS analysis  while managing their interdependencies. For example, the vast majority of tools that support singular repetitive tasks that can be run in parallel (mapping, filtering, etc.), rarely provide an easy solution for the integration of these tasks into a complicated multi-dimensional workflow. As such, few softwares allow users to efficiently run custom made pipelines on the same server on which the data is stored long term. For example, Galaxy, the most widely used open-source platform for data analysis has a powerful and intuitive web-based front-end interface. Nevertheless, users are required to upload files and are often limited by various regulations including maximum job submissions, wall time and storage space. Other tools such as HTStation [16] require scientists to continuously follow the job statuses and manually manipulate files and keys between different steps. These iterative and error-prone processes, which, *de facto,* cannot be referred to as pipelines, are cumbersome and time-consuming.

To address some of the above-discussed issues, we present NEAT, a framework developed to help manage ChIPseq and RNAseq pipelines in a robust, reproducible and user-friendly manner. NEAT offers several automated modules (unzip, rename, QC, chiprx, map, filter, peakcalling, creation of wig files, etc) that can be run through double-clickable icons from any desktop or laptop, an interface that not only facilitates the analysis of NGS data, but that makes it accessible to non-expert users. Furthermore, NEAT includes downstream applications that allow users to effortlessly explore NGS data using a graphical user interface (GUI) display. In summary, we believe that NEAT will help biologists as well as established bioinformaticians create, manage and analyze complex NGS pipelines, as well as assess NGS data within 24 h of the sequencing run completion through a simple GUI.

## Implementation

We have created an NGS framework under the UNIX operating system called NEAT that can easily be run either through the command line or through a graphical user interface (GUI). NEAT is a modular, reliable and user-friendly framework that allows users to build both ChIPseq and RNAseq pipeline using plain words (‘*map*’ will map and so on). NEAT is completely automated and supports users in the analysis of NGS data by managing all jobs and their dependencies from a single, centralized file. NEAT is designed to be run by scientists with no programming experience and as such, pipelines can be build and managed using double-clickable executables on a simple laptop. On the other hand, its modular architecture allows advanced users to easily customize NEAT to their own needs. In additional, NEAT can be implemented in the vast majority of institutions (compatible with LSF and PBS) regardless of rules and regulations as all cluster parameters including queuing priority, node allocation, number of CPUs and wall time can be parameterized from a single file.

**Fig. 1:**
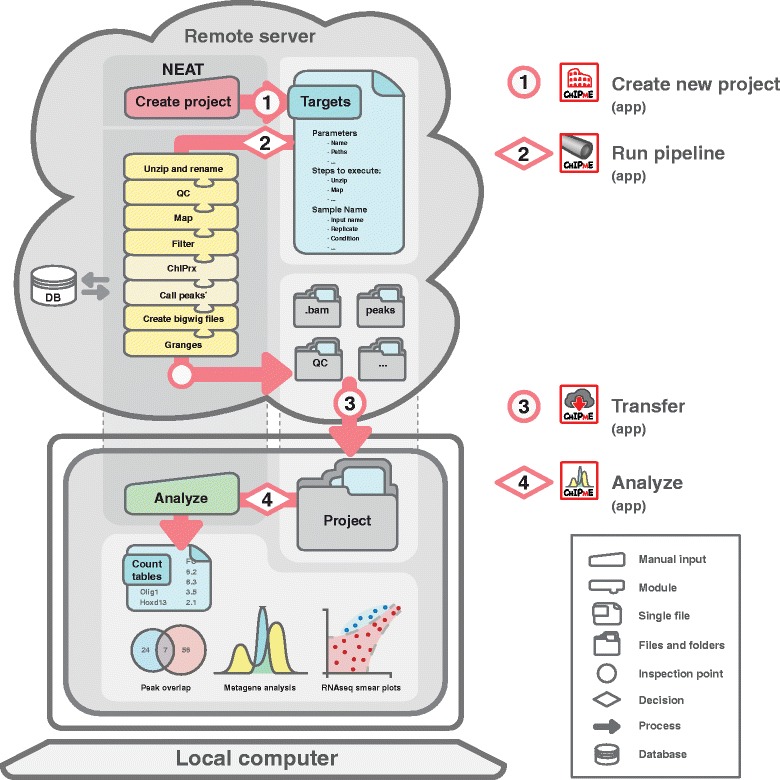
NEAT architecture. NGS data can be analyzed using NEAT in less than a day. Users follow a logical 4-step process, including the creation of a new project, running the pipeline on a remote server or in the cloud, transferring the data to a local computer and proceeding to the analysis. ^*^depicts modules that are restricted to ChIPseq experiments. The modules depicted reflect a non-exhaustive list. Left-hand figure represents a conceptual framework; Right hand icons represent the double-clickable executables that run the different processes

After having installed NEAT using the ‘*Install.app*’ module (Additional files 1 and 2), the analysis consists of a 4-step process, each of which has a unique GUI: (1) create a new project; (2) run NEAT; (3) download data to local computer; (4) run additional analyses (Fig. 1 and Additional files 1 and 2). Although all steps can be run either on a remote server or on a local computer, step (2) requires high computational power and we therefore suggest running it through the GUI, which will launch the pipeline on the remote high capacity cluster specified by the user. Sections (1), (3) and (4) can be run locally without the need for internet access, which is an advantage for users that are uncomfortable with cloud computing or when traveling.

The four steps of the NEAT framework are described below. In addition, step-by-step tutorials can be found in the supplemental material (Additional files 1 and 2). The tutorials allow users to follow through an entire NGS analysis using a provided test data set. The test datasets, which are either H3K4me3 ChIPseq data or RNAseq data from mouse embryonic stem cells, have been truncated such that the entire analysis should take less than two hours. Running the test data will also ensure NEAT and its dependencies (packages, scripts, etc) are properly installed before submitting large, memory-savvy analyses.

### Step 1: Creating a NEAT project

The first step of the NEAT framework is to build a new project. This can be done through the ‘*New Project*’ application (Fig. 1 and Additional files 1 and 2), which will prompt users to enter some details including the directory the project will be created in and the name of the project. Once executed, the user will be asked to fill in the foremost important step of NEAT: the *Targets.txt* file.

The *Targets.txt* file is the most important piece of NEAT and users are expected to invest the time and effort to ensure all paths and parameters exist and are correctly set. It is worth noting that once set, most of these parameters will not change on a specific computer cluster (users from the same institute will use the same parameters). We therefore suggest that more advanced users modify the *original Targets.txt* template file (Additional files 1 and 2), which is used as template each time a new project is created. This will significantly ease the process of building new projects and will minimize errors due to inexistent files or wrong paths. For down stream analysis of NEAT projects (see step 4), several widely used database names can be found in the *Species_specificities.txt* file for reference (Additional files 1 and 2).

**Fig. 2:**
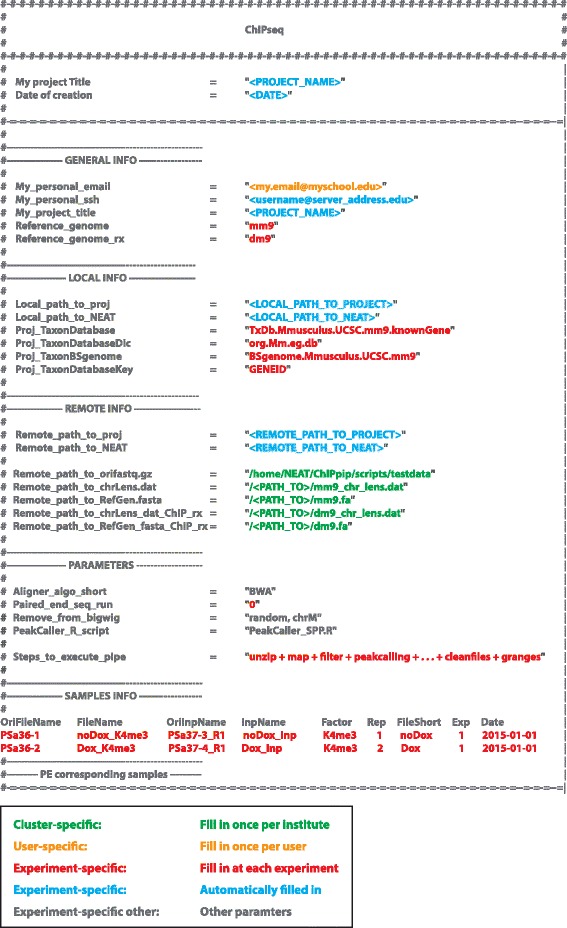
The Target.txt file. NEAT is centralized around a single text file (*Targets.txt*) containing all required information including sample names, inputs, their relationships, virtual paths to reference genomes, parameters, alignment and peak calling algorithms. Many of the settings are either automatically filled in when a new project is created or need to be filled in only once. The color code helps understand which parameters are specific to the cluster (green); the user (orange); the experiment (red and blue) or different parameters (grey)

While the upper portion of the *Targets.txt* file sets up the backbone of NEAT, the bottom portion contains the details of the experimental setup including the names of the compressed fastq files (usually provided by the sequencing core), the names that the users would like to give to the samples, their relationship (replicate, sample to input, etc) and antibody specificities. For paired-end runs, it is important to note that the sample name of the reverse-reads needs to be consistent with the forward-reads sample name, followed by ‘_R2’ (underscore R2). For example, if the ‘*FileName*’ of the forward reads is ‘PSa36-1_Dox_K4me3’, the corresponding reverse read file should be named ‘PSa36-1_Dox_K4me3_R2’. In addition, the reverse-reads file information should be set below the *‘PE corresponding samples’* mark at the bottom of the *Targets.txt* file instead of the *‘SAMPLES INFO’* section (Fig. 2 and Additional files 1 and 2).

Finally, in addition to containing the data processed by the pipeline, most importantly, the *Targets.txt* file contains the building blocks of the pipeline. These blocks are specified under the ‘*Steps_to_execute_pipe*’ and can be written in plain English words, e.g. ‘unzip’, ‘map’, ‘filter’ etc. The different default building blocks are described below. As NEAT uses exact word matching, users that do not want to run a given block are free to delete it or rename it (for example as 'chiprs_NO').

#### Unzip

The ‘*unzip*’ module will unzip, rename and store fatsq files in a newly created folder within the project folder. Although this strategy can seem cumbersome for space issues, it allows systematic storage of backups without manipulating the original compressed file, which helps organize and keep track of the sequencing runs.

Sequencing cores use different compression formats. For this reason, users can specify the file extension and the unzip command in the *AdvancedSettings.txt* file (Additional files 1 and 2). This module will unzip the compressed files found in the directory specified in the ‘*Remote_path_to_orifastq_gz*’ parameter and which names are found in the ‘*OriFileName*’ column of the *Targets.txt* file, and will rename them according to the users setup in the ‘*FileName*’. All files will be stored in the newly created ‘*fatsq*’ folder (Additional file 3).

#### QC

The ‘*QC*’ module uses the R systemPipeR package (Girke T. (2014) systemPipeR: NGS workflow and report generation environment. URL https://github.com/tgirke/systemPipeR) to provide a variety of high quality control outputs including per cycle quality box plots, base proportion, relative k-mer diversity, length and occurrence distribution of reads, number of reads above quality cutoffs and mean quality distribution. The ‘*QC*’ building block, together with the ‘*GRanges*’ modules (see below) are the rare exception that require the installation of external R packages. Additional information on package installation can be found in the tutorials (Additional files 4, 1 and 2).

#### ChIPrx

ChIPrx [17] is a cutting edge normalization method for ChIPseq that performs genome-wide quantitative comparisons using a defined quantity of an exogenous epigenome, e.g. a spike-in control. The detailed algorithm of ChIP-RX has been implemented as previously published [17]. For the sake of consistency, the same mapping and filtering parameters will be used for both the alignment of the standard and the spike-in epigenome. If no spike-in controls are used, all ChIP-RX parameters can be dashed (‘-’).

#### Map

The ‘*map*’ module maps reads using either bwa [18] or bowtie [19]. For RNAseq projects, the splice-aware, bowtie-based Tophat [20] algorithm is preferred. The standard parameters for either algorithm can be modified in the *AdvancedSettings.txt* file, including maximum number of gaps, gap extension, maximum edit distance, number of threads, mismatch and gap penalty, etc. Additional mapping algorithms can easily be implemented by advanced users (Additional files 1 and 2).

#### Filter

The ‘*filter*’ module allows the user to specify filtering parameters (*AdvancedSettings.txt*) including how to manage duplicate reads, minimum and maximum size of fragments, etc. This module uses the samtools [12, 21] *view*, *sort*, *rmdup* and *index* functions.

#### Peakcalling

The ‘*peakcalling*’ module specifies the algorithm used to call peaks. NEAT has two well-established peak calling methods built-in by default, including MACS (PeakCaller_MACS.R) [22] and SPP (PeakCaller_SPP.R) [23]. It is worth noting that given that NEAT is open source and very versatile, it is easy for advanced users to implement their own peak calling algorithm as an R code (Additional files 1 and 2).

#### Cleanfiles

Given different mapping algorithms have distinct outputs, the ‘*cleanfiles*’ module helps reorganize and store the different .bam and .bai files before proceeding to downstream analysis. This allows advanced users to implement their own mapping algorithms while still taking advantage of NEAT’s EDA modules.

#### GRanges

The ‘*GRanges’* module creates significantly smaller *GRanges* objects (compared to bam files), which are necessary for downstream analysis including identification of differentially regulated genes (RNAseq) and metagenomic analyses (ChIPseq). This eases and increases the efficiency of file transfer, file sharing and consolidation of projects. In addition, the ‘*GRanges*’ module creates small size wiggle files (.wig files). Wiggle files can be loaded and visualized in various genome browsers including IGV [24, 25]. The compression of the file is driven in part by the binning of the data across the genome. The bin size, which is in base pair units, can be customized in the *AdvancedSettings.txt* file.

### Step 2: Running NEAT

After building a pipeline using the easy one-word method in the ‘*Steps_to_execute_pipe*’ line of the *Targets.txt* file, non-expert users can run the workflow using the applescript double-clickable executable (Fig. 1 and Additional files 1 and 2). More advanced users can run it through the command line (Fig. 1 and Additional files 1 and 2). The executable will prompt users to identify which project they want to run before opening a terminal and asking them (twice) to enter their ssh password. This will allow NEAT to access and run the pipeline on the computationally efficient remote cluster. Once entered, NEAT automatically manages job submission, queuing and dependencies. A detailed explanation on how to follow the pipeline and a step-by-step debugging support can be found in the tutorials (Additional files 1, 2 and 5). Moreover, users can decide to setup automatic emailing when the pipeline has completed. As a point of reference, running an exhaustive pipeline (unzip + QC + chiprx + map + filter + peakcalling + cleanfiles + granges) on data comprising 200–400 million reads should not take more than 10 to 15 h. The project architecture of a completed NEAT project on the remote server including the timing and location of files and folders can be found in Additional file 3.

### Step 3: Download a NEAT project from a remote server to a local computer

The core component of NEAT (step 2), which is the pipeline *per se*, is computationally demanding and is thus preferentially ran on a remote cluster. However, upon completion of the pipeline, users may prefer to view and analyze their data locally, e.g. on a desktop or a laptop. As mentioned above, NEAT can be used to create GRanges and wiggle files, which main advantage are their relatively small size compared to bam files (wig ~ 4–6 Mb; GRanges ~ 40–60 Mb; bam ~ 4–6 Gb). In addition, these files can easily and rapidly be shared by email or in batch using standard flash drives.

To download a NEAT project from a remote server to a local computer, users can run the ‘*Transfer.app*’ applescript double clickable executable (Fig. 1 and Additional files 1 and 2), which will automatically open a terminal window and start the process. Users will be prompted to locate the NEAT directory and the NEAT project. The ‘*Transfer.app*’ will use all the information found in the corresponding *Targets.txt* file to download the NEAT project from the remote server to the local computer. Users should be attentive as they will be asked to enter the corresponding ssh password several times. Downloading an entire project should not take more than a few minutes.

### Step 4: Exploratory data analysis using NEAT

Empirically, data visualization is an important milestone for wet-lab scientists. This step is often critical for deciding the direction to take for further experiments and computational biologists often underestimate its importance. As an effort to improve the turn around time of NGS datasets, NEAT supports users in the creation of wig files (see step 2) that can be visualized using various genome browsers including IGV [24, 25].

**Fig. 3:**
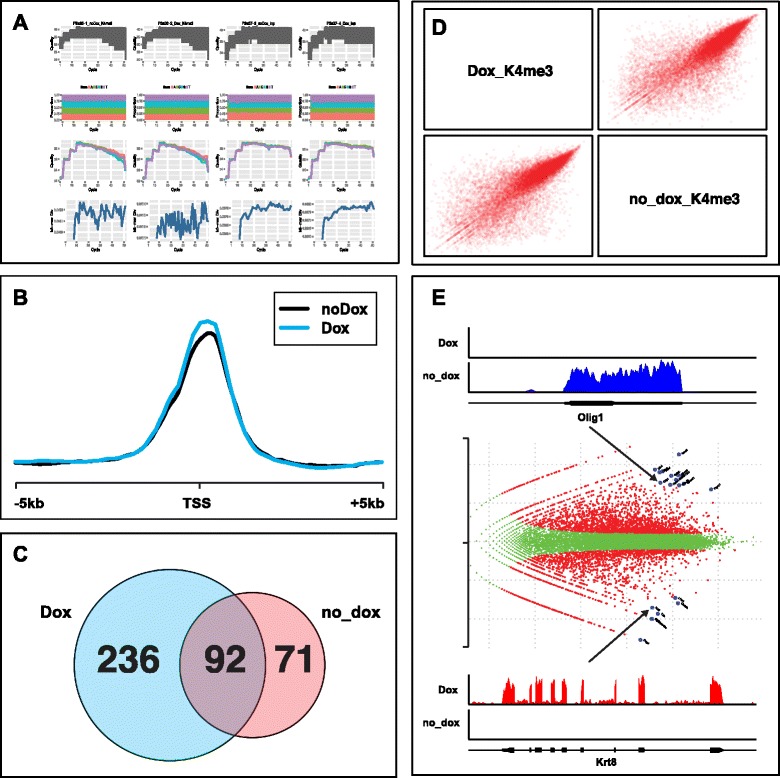
Output examples. NEAT outputs many files and graphs, some of which are depicted as examples. **a** Quality control of fastq files. **b** Metagene analysis (ChIPseq) of the test data around all TSSs. **c** Venn diagrams of peak overlaps. **d** Scatterplots for sample-to-sample comparison (RNAseq). **e** DEG smear plots with DEG highlighted and annotated. Above and below are two examples of DEG picked up by the pipeline

Section 4 of NEAT also contains tools for exploratory data analysis (EDA), which supports the creation of human-readable files including pdf graphs and count tables, which can be opened and analyzed in softwares such as excel. The tools that create these files require relatively small computational power, which allows users to experiment using a variety of different parameters ranging from cutoff values to DEG stringencies. The default EDA tools consist of [ChIPseq]: metagenomic analysis (feature-centric alignment of ChIPseq enrichments), count tables and peak overlap (if SPP was used as peak calling algorithm); [RNAseq] smear plots, DEG analysis, consolidated count tables, RPKM, Venn diagrams of gene overlap and GOrilla-compatible [26, 27] differentially expressed genes lists (Fig. 3).

It is worth noting that the metagenomic analysis in the ChIPseq module can be easily customizable. This tool allows users to visualize chromatin immunoprecipitation enrichments of various samples over specific features (contained in the *MartObject* folder; Additional files 1 and 2). For example, using the test dataset, users can explore enrichment of an epigenetic mark (K4me3) around all transcriptional start sites (TSSs) of the mouse genome. However, such analyses are not constrained to any particular region, nor to regions of similar length. By creating a simple bed file, users can assess enrichments over their preferred regions of interest. For example, users can visualize enrichments over all transcripts and/or enhancers. In such case, the length will be normalized throughout all regions. Any bed-formatted file can be used for the metagenomic module.

### Customizing NEAT modules

NEAT was developed as a user-friendly, intuitive and versatile tool. As such, care has been taken to allow users the ability to customize the pipeline for their own needs. This includes easy customizable mapping algorithms, mapping and filtering parameters, peak calling algorithms and metagenenomic features (TSS, transcripts, personal regions of interest, etc). In addition, more advanced users can efficiently develop novel modules as the code architecture has been written in a robust, logical, highly redundant and well-annotated manner. To add a new module, advanced users can simply duplicate an existing module and integrate their custom task into the script, usually consisting of a single line of code. The NEAT framework fully automates recurrent tasks such as batch job submissions, job dependencies, job queuing, error management, filing, etc., which greatly facilitates the creation of custom modules. Full support and step-by-step explanations to add customized modules can be found in the tutorials (Additional files 1, 2, 5, 6 and 7).

## Results

As this work presents a 'pipeline', tangible results are in the form of outputs (Fig. 3). Supporting arguments are in-line.

## Conclusion

Technological revolutions often drive and precede biological revolutions. The *omics* field has not been immune to this general rule. Such paradigm shifts are often followed by a period of great adaptation. For massively parallel sequencing, developing curriculums to educate scientists with the proper skill sets will require some time. Meanwhile, the life science community is in desperate need for tools to support scientists that have been trained prior to the sequencing of the human genome. Although NEAT is not intended to replace thorough bioinformatics analysis *per se*, we believe that it provides helpful tools to accompany scientists in the analysis of NGS data and allow them to rapidly apply standard exploratory data analysis methods to assess the quality of their experiments within 24 h of the sequencing run completion. Specifically, we strongly believe that providing wet-lab scientists with simple tools to facilitate rapid data visualization, which is a significant bottleneck for many users, will greatly benefit the community and will allow one to better plan and foresee biological experiments without the need to wait for thorough bioinformatics analysis.

NEAT was developed for a wide audience including scientists with no *a* priori programming knowledge. To this end, although NEAT should be self explanatory (double-clickable application based), it comes with step-by-step tutorials as well as two test datasets that will enable novice users to follow through and reproduce entire ChIPseq and RNAseq workflows. In addition, given the wide diversity of interests in the life sciences, NEAT has been developed to be versatile, easily customizable and applicable to a wide variety of different genomes. Finally, the modular structure of NEAT allows advanced users and computational core facilities to easily add and modify tasks, customize settings and comply with internal rules and regulations with minimal footprint to their existing server architecture. Taken together, we believe NEAT will be of general interest and has the potential to be widely adopted for its versatility and ease of use.

NEAT is an open-source software under an MIT license. NEAT, including tutorials and test data, is publicly available on GitHub (https://github.com/pschorderet/NEAT).

## Availability and requirements

Project home page: https://github.com/pschorderet/NEAT

Operating system: Mac OSx

Programming language: Perl, R, Applescript

License: NEAT is an open-source software under an MIT license

## Additional files

Additional file 1:
**Quick guide ChIPseq.** Step-by-step guide for the analysis of ChIPseq datasets, including the provided test dataset. (PDF 834 kb) Additional file 2:
**Quick guide RNAseq.** Step-by-step guide for the analysis of RNAseq datasets, including the provided test dataset. (PDF 843 kb) Additional file 3:
**Code architecture.** Architecture of the provided NEAT ChIPseq project after completion of all steps on the remote server. The color code highlights which files are created during which step as well as where they are stored. (PDF 34 kb) Additional file 4:
**QC report.** QC report generated by NEAT when running the ChIP- and RNAseq test data set. (PDF 889 kb) Additional file 5:
**Quick guide to add custom modules.** Step-by-step guide for the addition of custom modules. (PDF 360 kb) Additional file 6:
**Code architecture schematic.** Example of a module’s architecture in NEAT. The left part schematically represents the different steps that constitute each module. The right part represents some example code and how it is imbricated. The example reflects NEAT run on a torque manager system (qsub/PBS) though NEAT can be run on other systems as well including LSF clsuters (bsub). (PDF 960 kb) Additional file 7:
**Code architecture.** Code architecture for the additional of custom modules. Custom code should replace the red font (make sure the loop is correct depending on whether it is a ChIPseq or RNAseq module). The module backbone as well as the submission procedures are robust, highly repetitive and will automatically manage job submission and queuing. (PDF 458 kb)
