# Supplementary material for: NEAT: a framework for building fully automated NGS pipelines and analyses
Source: BMC Bioinformatics. 2016 Feb 1;17:53. doi: 10.1186/s12859-016-0902-3 (PMC4736651; doi:10.1186/s12859-016-0902-3)
Supplement: Additional file 1: — Quick guide ChIPseq. Step-by-step guide for the analysis of ChIPseq datasets, including the provided test dataset. (PDF 834 kb) [file 12859_2016_902_MOESM1_ESM.pdf]

Quick Guide

NEAT for ChIPseq

NGS pipelines made easy

Patrick Schorderet

Patrick.schorderet@molbio.mgh.harvard.edu

June 2015

1 INTRODUCTION 4
2 NEAT 6
2.1 INTRODUCTION TO NEAT 6
2.2 INSTALL NEAT 7
3 NEAT PART 1 AND 2 8
3.1 BEFORE RUNNING NEAT FOR CHIPSEQ 8
3.2 RUNNING NEAT PART 1 8
3.2.1 Creating a new ChIPpip project 8
3.2.1.1 Filling in the Targets.txt file 9
3.3 RUNNING NEAT PART 2 12
4 NEAT PART 3 AND 4 14
4.1 RUNNING NEAT PART 3 14
4.1.1 Step 1: Download a ChIPpip project 14
4.2 RUNNING NEAT PART 4 15
4.2.1 Step 2: Run a ChIPmE analysis 15
4.2.2 Mart objects 15
4.3 OUTPUTS 16
4.3.1 Logs 16
4.3.2 Count tables 16
4.3.3 Metagene plots 17
4.3.4 Wig files 17
4.4 ADVANCED SETTINGS 18
4.4.1 Custom mart objects 18
4.4.2 Bam files and GRanges 18
4.4.3 Consolidating projects 18
5 VERSION INFORMATION AND REQUIRED PACKAGES 19
6 REPORTED BUGS 20
7 FUNDING 20
8 REFERENCES 21

1 Introduction

The ***NE***xt generation ***A***nalysis ***T***oolbox (NEAT) is a perl/R package that supports users during the analysis of next generation sequencing (NGS) data.

NEAT provides an easy, rapid and reproducible exploratory data analysis (EDA) tool that allows users to assess their data in less than 24 hours (based on a 200mio read Highseq run). NEAT was developed in four main sections. The two first sections (creating and running a pipeline) helps users with jobs that are computationally demanding (mapping, filtering, etc). The two last sections consist of analyses that can be run locally (on a desktop computer). All four sections are standalone applications.

This quick guide is intended for users who want to run a quick analysis and are comfortable using the default parameters and algorithms. Please refer to the complete guide for more details on in depth analysis.

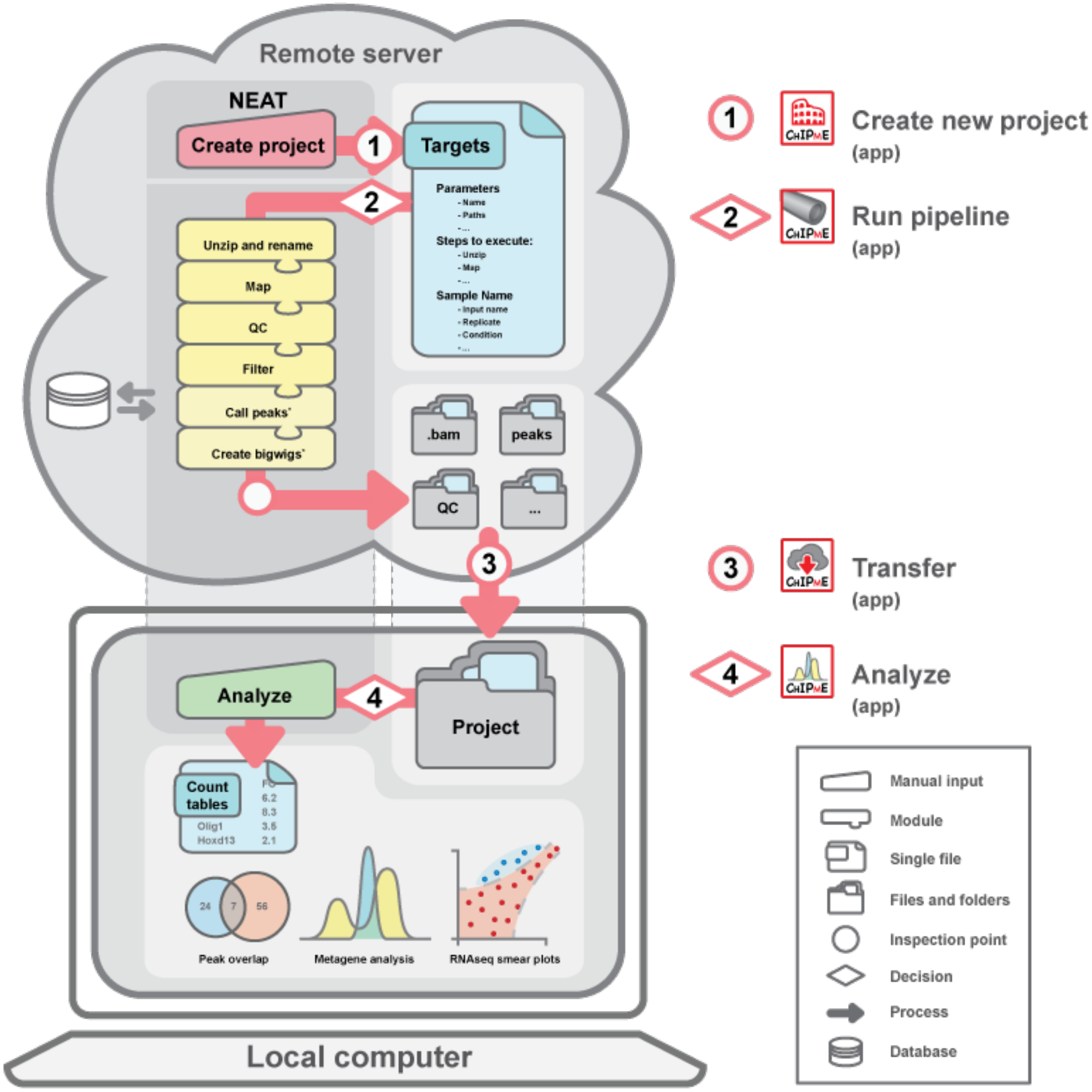

Fig.1 NEAT architecture. NGS data can be analyzed using NEAT in less than a day. Users follow a logical 4-step process, including the creation of a new project, running the pipeline on a remote server or in the cloud, transferring the data to a local computer and proceeding to the analysis.

2 NEAT

2.1 Introduction to NEAT

A central feature of NEAT is its ability to perform repetitive tasks on complex sample setups while managing batch submissions and cluster queuing. NEAT can easily be implemented in most institutions with limited to no programming experience. The workflow has been designed to efficiently run on a computer cluster using a distributed resource manager such as TORQUE (qsub commands) or LSF (bsub commands). NEAT has been developed by and for wet-lab scientists as well as bioinformaticiens to ensure user-friendliness, management of complicated experimental setups and reproducibility in the big data era.

To start using NEAT, please follow the tutorial. This will walk you through the analysis of a small test dataset using your own computer cluster. This will also ensure NEAT and its dependencies are correctly installed before submitting large, memory-savvy analysis.

All fastq files from the test data have been subsetted to ca. 15’000 reads. This data comes from an unpublished 50bp single end (SE) sequencing experiment although NEAT can deal with paired-end (PE) sequencing as well. For more information on the test data provided in this tutorial, please read below.

Although this quick guide is intended for scientists with no programming experience, the pipeline ran by NEAT will be launched on a remote server. Users therefore require an SSH access with a username and a password. Please refer to your system administrator to obtain such credentials.

2.2 Install NEAT

To install NEAT, download the NEAT repository (https://github.com/pschorderet/NEAT) from GitHub to any directory on your computer.

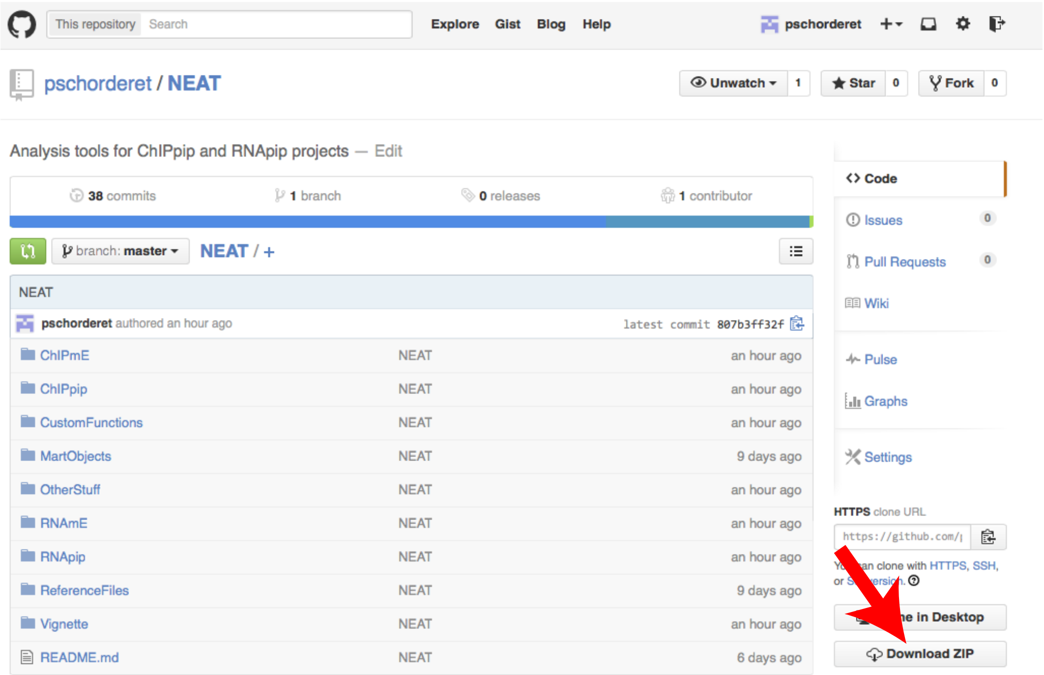

This tutorial assumes the *NEAT/* directory was saved to the user's desktop on a local computer (*~/Desktop*) and that it will be saved to the home directory (*/home/*) on the remote server.

Make sure the folder is named *NEAT* and not *NEAT-master*. Run the *install* package and follow the instructions.

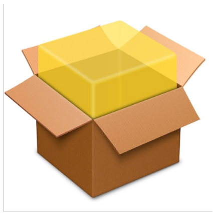

Enter your password and allow for the folders to transfer. This step installs all required R packages on your personal computer. Please ensure there are no errors before proceeding to the analysis.

3 NEAT part 1 and 2

3.1 Before running NEAT for ChIPseq

Please make sure all R packages required for NEAT are installed on your computer (refer to the R manual) prior to running NEAT. Refer to the *Version information and required packages* section below for more information. In addition, all four NEAT standalone applications run through *Automator*, a MacOS application standardly installed on most modern Apple computers, please make sure it is installed on your computer.

Finally, ensure your *Terminal* software is closed before launching NEAT.

3.2 Running NEAT part 1

3.2.1 Creating a new ChIPpip project

The first step to run NEAT on ChIPseq data is to create a new ChIPpip project. Double-click the *1_NewProject.app* found in the NEAT directory *~/Desktop/NEAT/ChIPmE/* and follow the prompts.

We advise to save projects in other directories than the NEAT directory itself so that you can update NEAT by dragging and dropping the entire NEAT folder without compromising older projects.

3.2.1.1 Filling in the Targets.txt file

Creating a new project (named by default MY_NEW_CHIP_PROJECT) should create the central part of NEAT: the Targets.txt file. The Targets.txt file can be found in *~/MY_NEW_CHIP_PROJECT/DataStructure/* and is the backbone of NEAT. It contains all the information specific to your experiment and your computer cluster, including the names of files, the paths to the reference genomes, the steps to execute, the name of your samples, their relationships, etc.

IMPORTANT: Sample names including inputs and fastq files cannot start with the letter 'n' (small or capital) as this is the universal perl symbol for carriage return. They can also not contain '_R2' other than to name the corresponding paired-end samples (see files).

The *Targets.txt* file is the most important piece of NEAT and users are expected to invest the time and effort to ensure all paths and parameters exist and are correctly set. However, once set, most of these parameters will not change on a specific computer cluster (users from the same institute will use the same paths). We therefore suggest that more advanced users modify the *original* Targets.txt template file (see below).

All parameters of the Targets file should be self-explanatory. Below is a brief summary:

My_personal_email	:	If users would like to be notified by emailed when the cluster has finished. This will only work if your computer cluster has activated the emailing feature (please check with system administrator). To ensure servers are not overwhelmed by email services, ChIPpip is configured in such a way as to notify users only if the pipeline has terminated properly (with no error).

Users may change this parameter by modifying the QSUB_header.sh template file found in *./ChIPpip/scripts/*.

My_personal_ssh : SSH parameters. Refer to your computer core facility

My_project_title : This is the name of the folder of your project on the remote server [automatically generated by ChIPpipCreateNewProject].

Reference_genome : The genome your data will be aligned to. Make sure your core faility has this genome reference installed on your cluster and that the extensions of the files are '.fa'.

Reference_genome_rx : Spike-in reference genome (ref. Orlando *et al.* Cell Reports, 2014)

Local_path_to_proj : [Automatically generated by ChIPpipCreateNewProject].

Local_path_to_NEAT : [Automatically generated by ChIPpipCreateNewProject].

Proj_TaxonDatabase : See NEAT for RNAseq.

Proj_TaxonDatabaseDic : See NEAT for RNAseq.

Proj_TaxonDatabaseDic : BSgenome (f.ex: BSgenome.Mmusculus.UCSC.mm9). Available genomes can be found on the bioconductor website: http://master.bioconductor.org/packages/release/bioc/html/BSgenome.html

Remote_path_to_proj : Full path to your project folder (without the project name) [automatically generated].

Remote_path_to_NEAT : Full path to your NEAT folder. Note that in our example, we have created our project within the ChIPpip folder itself, but users can freely decide to create a dedicated folder for all of their ChIPpip projects.

Remote_path_to_orifastq_gz Full path to where your compressed .fastq.gz files are. Usually, your sequencing core facility will let you know where they store these files. Note that all .fastq.gz files can be kept in a single location, they do not need to be copied to your folder.

Remote_path_to_chrLens_dat Path to a .dat file containing chromosome information for your reference genome. Refer to your computer core facility.

Remote_path_to_RefGen_fasta Path to folder containing your reference genome files. When using BWA, this folder will contain the indexes (**.fa.** files) as well as the fasta file (*.fa, .fai*). When using BOWTIE, this folder will contain the indexes (**.ebwt* files) as well as the fasta file (*.fa, .fai*). Refer to your computer core facility.

Remote_path_to_chrLens_dat_ChIP_rx: See above.

Remote_path_to_RefGen_fasta_ChIP_rx: See above.

Aligner_algo_short : "BWA" or "BOWTIE" for standard alignment. If other algorithms are used, modify the *AdvancedSettings.txt* file accordingly.

Paired_end_seq_run	:	"0" for single end sequencing. "1" for paired end sequencing.
PeakCaller_R_script	:	NEAT comes with two preinstalled algorithms: SPP (PeakCaller_SPP.R) and MACS (PeakCaller_MACS.R). For advanced users, any peakcalling algorithm can be used. Wrap your algorithm in a .R files and enter the name of the file in the PeakCaller_R_script parameter.
Steps_to_execute_pipe	:	Users can choose from the following tasks<br>• *Unzip*<br>• *QC*<br>• *ChIPrx*<br>• *Map*<br>• *Filter*<br>• *Peakcalling*<br>• *Cleanfolders*<br>• *GRanges*<br><br>If you do not want to run all of these, simply delete them for the Targets.txt or rename them. Once ran, ChIPpip will change the value of these from '*unzip*' to '*unzip_DONE*'. Obviously, a certain hierarchy has to be followed, e.g. attempting to filter reads without having previously mapped them (in the same run or in a previous run) will not work. Note that '*qc*' requires Thomas Girke's systemPipeR package; map requires bwa; the default *peacalling* requires the R package SPP; *filter* requires samtools; *Granges* requires various well-established R packages. Refer below for exact requirements.
Sample naming	:	For single-end (SE) runs, fill in the '*SAMPLES INFO*' section. Characters including spaces, dollar signs and other well-known special characters should not be used. Underscores should be the reference character to delimit words. For paired-end sequencing runs, in addition to filling in the first section, fill in the '*PE CORRESPONDING SECTION*', which correspond to the .fastq files containing the reads from the reverse strand. The names of these files need to be identical to the exception of adding a '*_R2*' at the end of the name. For example, if the first file name is '*PSa29-5_noDox*, the corresponding reverse strand file will be named '*PSa29-5_noDox_R2*'. The order in which the files appear also needs to be identical in both sections. Please refer to the example Targets.txt file for more information.

Please modify the *Targets.txt* file according to your needs. To modify the Targets.txt file, we suggest users get accustomed to using plain text editors such as TextWrangler as it will avoid including spaces and special characters. In addition, it is worth making sure that the parameters in the *AdvancedSettings.txt* files are

correct, especially the unzip command and extension as well as the alignment command (BWA vs Bowtie).

The paths to the reference genomes should be obtained from your computer core facility (system administrator), as they are the ones maintaining these up to date. Note here that the reference genome files should have an '*.fa*' extension (e.g. mm9.fa). Please make sure that your core has named these files accordingly as any other extension will lead to a prematurely arrest of the pipeline.

In addition, *#Remote_path_to_RefGen.fasta* refers to the path to the file *<reference_genome>.fa* and not to the folder in which '*.fa*' files are located. This is different than in RNApip.

To avoid repeating these steps at each new NEAT project creation, we suggest you modify the *original Targets.txt* file that is used as template when creating a new project. This file can be found in *~/NEAT/ChIPpip/scripts/NewChIPpipProject/DataStructure/Targets.txt*.

3.3 Running NEAT part 2

Once the *Targets.txt* file is correctly set up, users can run the *2_Run_NEAT.app*.

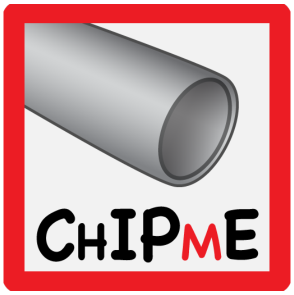

This script will execute the tasks specified in the *Targets.txt* file.

After asking the user to enter his/her ssh password (twice), this will launch the pipeline and will prompt a summary of the user's parameters. NEAT automatically manages all creations and batch submissions of jobs, dependencies, ordering of files, queuing, etc. If the cluster is using TORQUE, the processes can be followed on the terminal using the ***qstat (or bjobs)*** command (type ***qstat (or bjobs)*** in your terminal) after ssh-ing into your remote cluster. To ssh into a remote cluster, type in the following commands into a terminal window replacing your username and server address:

[MY_COMPUTE~]$ ***ssh username@serveradress.edu***

password

[username@setrveradress ~]$

Type ***qstat (or bjobs)*** to follow the status of the pipeline. Briefly, *Q* stands for queuing, *R* for running, *E* for exiting and *H* for holding.

Once the pipeline has finished, it will notify users of its status by email (if applicable).

The mock data provided as a test example should take no more than one hour to run, usually a lot less.

4 NEAT part 3 and 4

4.1 Running NEAT part 3

4.1.1 Step 1: Download a ChIPpip project

To transfer a NEAT project from a remote server to a local computer, double click the ChIPmE 3_*Transfer.app*.

Users will be prompted to locate the NEAT directory and the location they want to save their ChIPpip project. In this example, the *NEAT* directory is on the desktop.

The *3_Transfer.app* will use all the information found in the Targets file to download the ChIPpip project from the remote server to your local computer. Please be attentive as users will need to enter their ssh password several times.

Downloading an entire project should not take more than a few minutes.

4.2 Running NEAT part 4

4.2.1 Step 2: Run a ChIPmE analysis

Once the project has been downloaded, users can run the proper ChIPmE analysis. To this end, double click the NEAT *4_Analyze.app*.

Users will be prompted to locate the NEAT folder (saved on the desktop in this example). Users will also need to choose a mart object (see below). Finally, users will be asked to locate the ChIPpip folder (the one just downloaded). In this example, the ChIPpip project was downloaded to the desktop. Running the analysis using the test data should not take more than a couple minutes.

4.2.2 Mart objects

Mart objects are .bed files of regions of interest to align data to. Examples of mart objects are transcriptional start sites (TSS), all transcripts, enhancers, etc. Some mart objects are provided as part of the NEAT package in the MartObject folder. For this example, the data will be aligned over all TSS. The provided mart object (*mm9_TSS_10kb.bed*) is comprises of 10kb around all TSS of the mouse genome. Please note here that care should be brought to match the mart objects with the

reference genome initially used. In this example, the data was mapped to the mouse mm9 genome, hence the *mm9_TSS_10kb.bed*. Several parameters can be set before running the analysis including *binNumber*, *strand*, *runmeank*, *Venn* and *normInp*. Values are set as a reference, but we suggest users experiment to find the best values for their own need.

4.3 Outputs

4.3.1 Logs

Each time ChIPmE is run, a log file is created and named using the date and time. This file is save in the *~/MY_NEW_CHIP_PROJECT/logs/* directory. We strongly encourage users to initially look at these files, as any error that might have occurred will be saved there. Usually, if no error is prompted from the terminal, ChIPmE has terminated correctly. Also, please note that if there are unrecognized chromosome names such as random chromosomes, warning messages will appear. In the test data, there are 50 or more warnings. These can usually be disregarded.

4.3.2 Count tables

ChIPmE will generate count tables that should be self-explanatory. In brief, rows correspond to a mart object line and columns correspond to bins. Please note here that bins will be of same length for centered mart objects (for example TSSs) and will have names corresponding to base pairs, but will be of varied length for non-centered objects (for example transcripts), hence the columns will arbitrarily be named V1, V2, …, VN. Users should not worry about this to interpret graphs as it is accounted for by normalizing the values per bin by the bin length.

4.3.3 Metagene plots

ChIPmE generates pdf plots that are saved in *~/MY_NEW_CHIP_PROJECT/plots/*. Below is an examples of the plot generated for K4me3 over TSSs.

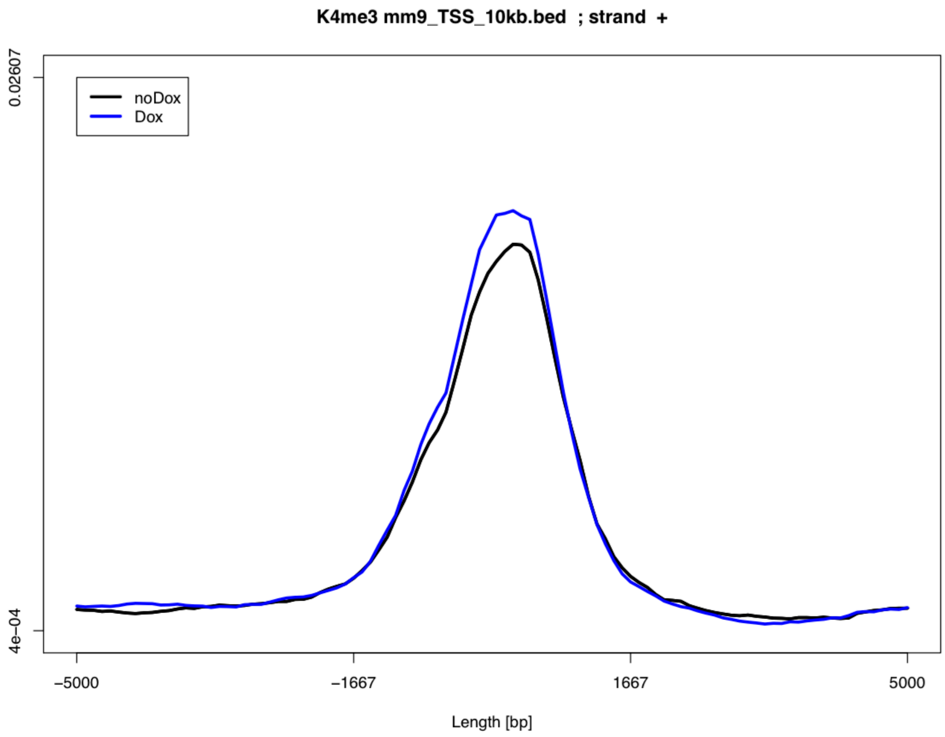

4.3.4 Wig files

GRanges and .wig files are created in the GRanges step (see above). Wig files can be directly uploaded to IGV for rapid and convenient visualization.

4.4 Advanced settings

4.4.1 Custom mart objects

Custom mart objects can easily be created with your favorites genes/regions. The files are simple .bed files that can either be manually or automatically created using various online tools or can be directly downloaded from genome browsers such as USCS or Ensembl. ChIPmE has been developed to ensure consistency between and within labs. In addition the relative small size of these bed files makes it easy to email/share them. We therefore suggest keeping an up-to-date, centralized folder containing all recurrent mart object files.

4.4.2 Bam files and GRanges

Bam files are generated during the initial phase (step 2) but are not automatically downloaded to the local computer. Rather, the GRanges files, which are often two orders of magnitude smaller in size (respectively several Gb vs tens of Mb) are dowlonaded. If required, bam files are always stored in the 'bam' folder on the remote server.

4.4.3 Consolidating projects

Consolidating projects is very easy. Users who intend to do so simply need to change the *Targets.txt* file and copy-paste the Granges objects (or bam files) from one folder to the other. Other files and folder can be left as is.

5 Version information and required packages

Program: bwa (alignment via Burrows-Wheeler transformation)
Version: 0.5.9-r16

Program: samtools (Tools for alignments in the SAM format)
Version: 0.1.18 (r982:295)

R version 3.1.0 (2014-04-10)
Platform: x86_64-redhat-linux-gnu (64-bit)

R packages (with dependencies) required to run ChIPpip:

- SPP (spp_1.11)
- systemPipeR (systemPipeR_0.99.0)
- ...

R packages (with dependencies) required to run ChIPmE:

- Rsamtools
- GenomicRanges
- GenomicAlignments
- caTools
- VennDiagram

locale:

[1] en_US.UTF-8/en_US.UTF-8/en_US.UTF-8/C/en_US.UTF-8/en_US.UTF-8

attached base packages:

[1] grid parallel stats4 stats graphics grDevices utils

[8] datasets methods base

other attached packages:

[1] VennDiagram_1.6.9 caTools_1.17.1 GenomicAlignments_1.2.1

[4] Rsamtools_1.18.2 Biostrings_2.34.1 XVector_0.6.0

[7] GenomicRanges_1.18.3 GenomeInfoDb_1.2.3 IRanges_2.0.1

[10] S4Vectors_0.4.0 BiocGenerics_0.12.1

loaded via a namespace (and not attached):
```
 [1] base64enc_0.1-2   BatchJobs_1.5     BBmisc_1.8        BiocParallel_1.0.0
 [5] bitops_1.0-6      brew_1.0-6        checkmate_1.5.0   codetools_0.2-9
 [9] DBI_0.3.1         digest_0.6.4      fail_1.2          foreach_1.4.2
[13] iterators_1.0.7   RSQLite_1.0.0     sendmailR_1.2-1   stringr_0.6.2
[17] tools_3.1.2       zlibbioc_1.12.0
```

6 Reported bugs

Refer to the complete guide for reported bugs

7 Funding

This pipeline was developed in part with funding from the Swiss National Science Foundation P300P3_158516 and in part through the R37 GM48405-21 grant awarded to Robert E Kingston

8 References

- Kharchenko P, Tolstorukov M & Park P. (2008) Design and analysis of ChIP-seq experiments for DNA-binding proteins. *Nature Biotechnology* **26**, 1351 - 1359
- Girke T. (2014) systemPipeR: NGS workflow and report generation environment. URL https://github. com/tgirke/systemPipeR.
- Li H. and Durbin R. (2009) Fast and accurate short read alignment with Burrows-Wheeler Transform. *Bioinformatics*, 25:1754-60.
- Michael Lawrence, Huber W, Pagès H, Aboyoun P, Carlson M, Gentleman R, Morgan MT, and Carey VJ. (2013) Software for computing and annotating genomic ranges. *PLoS Comput. Biol.*, 9(8):e1003118.
- Li H and Durbin R. (2009) Fast and accurate short read alignment with Burrows-Wheeler transform. *Bioinformatics*, 25(14): 1754–1760.
- Li H. (2013) Aligning sequence reads, clone sequences and assembly contigs with BWA-MEM. URL http: //arxiv.org/abs/1303.3997.
