# Supplementary material for: NEAT: a framework for building fully automated NGS pipelines and analyses
Source: BMC Bioinformatics. 2016 Feb 1;17:53. doi: 10.1186/s12859-016-0902-3 (PMC4736651; doi:10.1186/s12859-016-0902-3)
Supplement: Additional file 2: — Quick guide RNAseq. Step-by-step guide for the analysis of RNAseq datasets, including the provided test dataset. (PDF 843 kb) [file 12859_2016_902_MOESM2_ESM.pdf]

Quick Guide

NEAT for RNAseq

NGS pipelines made easy

Patrick Schorderet

Patrick.schorderet@molbio.mgh.harvard.edu

June 2015

1 INTRODUCTION 4

2 NEAT 6

2.1 INTRODUCTION TO NEAT 6

2.2 INSTALL NEAT 7

3 NEAT PART 1 AND 2 8

3.1 BEFORE RUNNING NEAT FOR RNASEQ 8

3.2 RUNNING NEAT PART 1 8

3.2.1 Creating a new RNApip project *8*

3.2.1.1 Filling in the Targets.txt file 9

3.3 RUNNING NEAT PART 2 12

4 NEAT PART 3 AND 4 13

4.1 RUNNING NEAT PART 3 13

4.1.1 Step 1: Download a RNApip project *13*

4.2 RUNNING NEAT PART 4 14

4.2.1 Step 2: Run a RNAmE analysis *14*

4.3 OUTPUTS 15

4.3.1 Logs *15*

4.3.2 Count tables and RPKM tables *15*

4.3.3 Differentially expressed gene plots *15*

4.4 ADVANCED SETTINGS 16

4.4.1 Bam files and GRanges *16*

4.4.2 Consolidating projects *17*

5 VERSION INFORMATION AND REQUIRED PACKAGES 18

6 REPORTED BUGS 19

7 FUNDING 19

8 REFERENCES 20

1 Introduction

The ***NE***xt generation ***A***nalysis ***T***oolbox (NEAT) is a perl/R package that supports users during the analysis of next generation sequencing (NGS) data.

NEAT provides an easy, rapid and reproducible exploratory data analysis (EDA) tool that allows users to assess their data in less than 24 hours (based on a 200mio read Highseq run). NEAT was developed in four main sections. The two first sections (creating and running a pipeline) helps users with jobs that are computationally demanding (mapping, filtering, etc). The two last sections consist of analyses that can be run locally (on a desktop computer). All four sections are standalone applications.

This quick guide is intended for users who want to run a quick analysis and are comfortable using the default parameters and algorithms. Please refer to the complete guide for more details on in depth analysis.

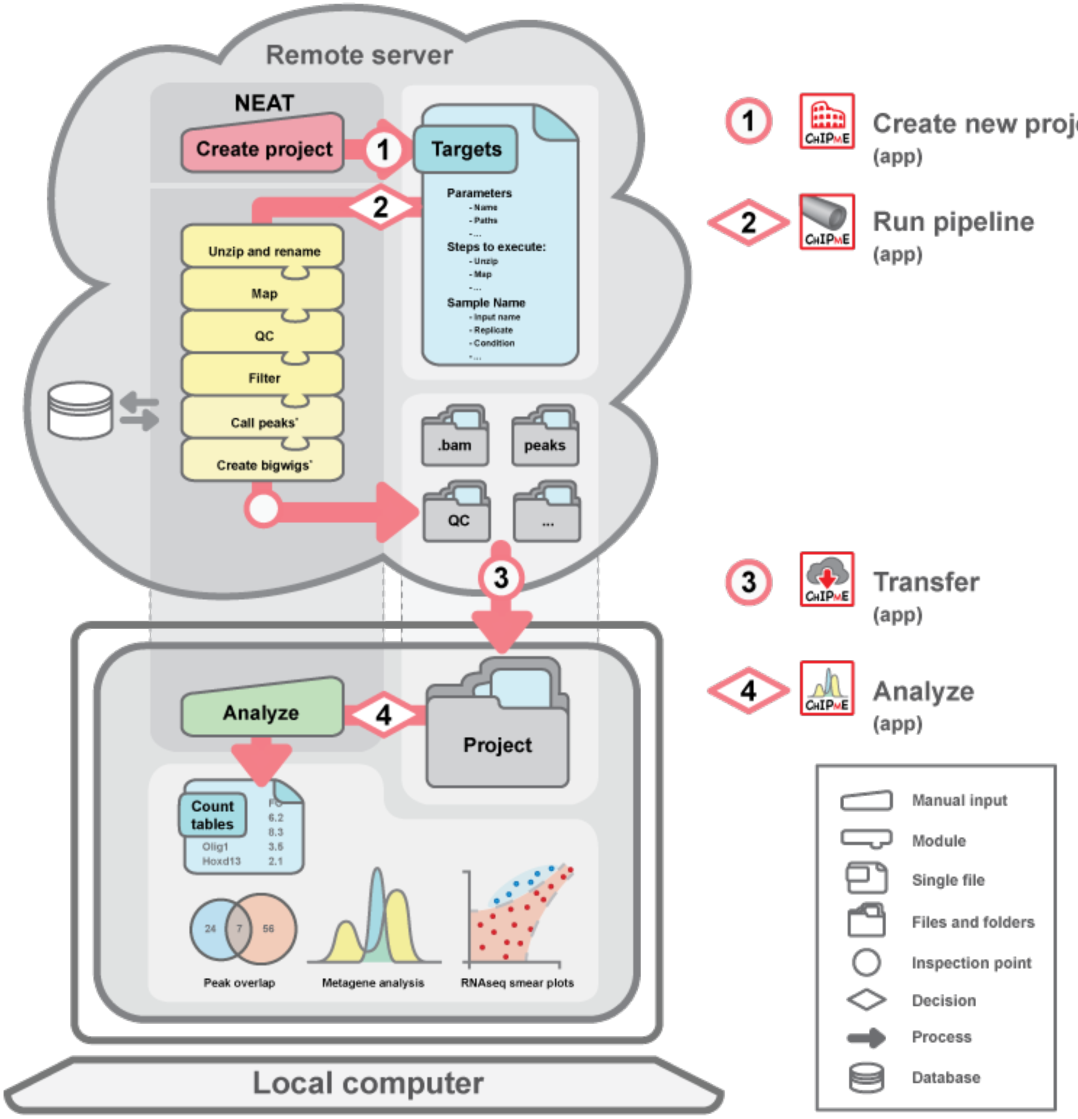

Fig.1 NEAT architecture. NGS data can be analyzed using NEAT in less than a day. Users follow a logical 4-step process, including the creation of a new project, running the pipeline on a remote server or in the cloud, transferring the data to a local computer and proceeding to the analysis.

2 NEAT

2.1 Introduction to NEAT

A central feature of NEAT is its ability to perform repetitive tasks on complex sample setups while managing batch submissions and cluster queuing. NEAT can easily be implemented in most institutions with limited to no programming experience. The workflow has been designed to efficiently run on a computer cluster using a distributed resource manager such as TORQUE (qsub commands) or LSF (bsub commands). NEAT has been developed by and for wet-lab scientists as well as bioinformaticiens to ensure user-friendliness, management of complicated experimental setups and reproducibility in the big data era.

To start using NEAT, please follow the tutorial. This will walk you through the analysis of a small test dataset using your own computer cluster. This will also ensure NEAT and its dependencies are correctly installed before submitting large, memory-savvy analysis.

All fastq files from the test data have been subsetted to ca. 15’000 reads. This data comes from an unpublished 50bp single end (SE) sequencing experiment although NEAT can deal with paired-end (PE) sequencing as well. For more information on the test data provided in this tutorial, please read below.

Although this quick guide is intended for scientists with no programming experience, the pipeline ran by NEAT will be launched on a remote server. Users therefore require an SSH access with a username and a password. Please refer to your system administrator to obtain such credentials.

2.2 Install NEAT

To install NEAT, download the NEAT repository (https://github.com/pschorderet/NEAT) from GitHub to any directory on your computer.

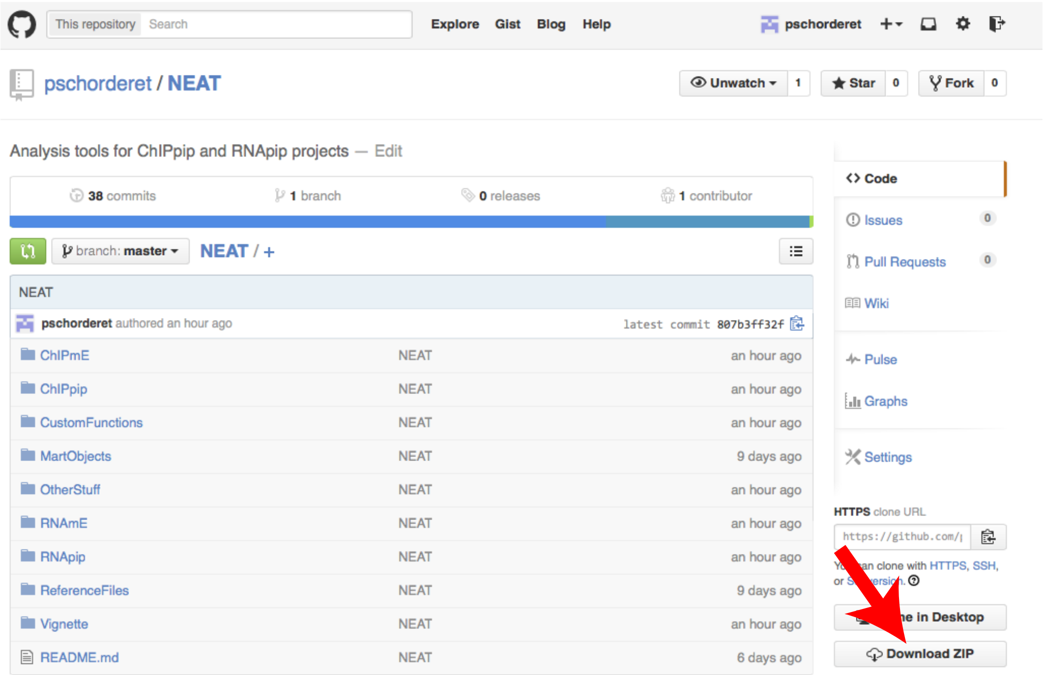

This tutorial assumes the *NEAT/* directory was saved to the user's desktop on a local computer (*~/Desktop*) and that it will be saved to the home directory (*/home/*) on the remote server.

Make sure the folder is named *NEAT* and not *NEAT-master*. Run the *install* package and follow the instructions.

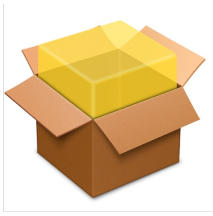

Enter your password and allow for the folders to transfer. This should also install all required R packages on your personal computer. Please ensure there are no errors before proceeding to the analysis.

3 NEAT part 1 and 2

3.1 Before running NEAT for RNAseq

Please make sure all R packages required for NEAT are installed on your computer (refer to the R manual) prior to running NEAT. Refer to the *Version information and required packages* section below for more information. In addition, all four NEAT standalone applications run through *Automator*, a MacOS application standardly installed on most modern Apple computers, please make sure it is installed on your computer.

Finally, ensure your *Terminal* software is closed before launching NEAT.

3.2 Running NEAT part 1

3.2.1 Creating a new RNApip project

The first step to run NEAT on RNAseq data is to create a new RNApip project. Double-click the *1_NewProject.app* found in the NEAT directory *~/Desktop/NEAT/*RNA*mE/* and follow the prompts.

We advise to save projects in other directories than the NEAT directory itself so that you can update NEAT by dragging and dropping the entire NEAT folder without compromising older projects.

3.2.1.1 Filling in the Targets.txt file

Creating a new project (named by default MY_NEW_RNA_PROJECT) should create the central part of NEAT: the Targets.txt file. The Targets.txt file can be found in *~/MY_NEW_RNA_PROJECT/DataStructure/* and is the backbone of NEAT. It contains all the information specific to your experiment and your computer cluster, including the names of files, the paths to the reference genomes, the steps to execute, the name of your samples, their relationships, etc.

IMPORTANT: Sample names including inputs and fastq files cannot start with the letter 'n' (small or capital) as this is the universal perl symbol for carriage return. They can also not contain '_R2' other than to name the corresponding paired-end samples (see files).

The *Targets.txt* file is the most important piece of NEAT and users are expected to invest the time and effort to ensure all paths and parameters exist and are correctly set. However, once set, most of these parameters will not change on a specific computer cluster (users from the same institute will use the same paths). We therefore suggest to more advanced users that they modify the *original* Targets.txt template file (see below).

All parameters of the Targets file should be self-explanatory. Below is a brief summary:

My_personal_email	:	If users would like to be notified by emailed when the cluster has finished. This will only work if your computer cluster has activated the emailing feature (please check with system administrator). To ensure servers are not overwhelmed by email services, RNApip is configured in such a way as to notify users only if the pipeline has terminated properly (with no error).

		Users may change this parameter by modifying the QSUB_header.sh template file found in *./RNApip/scripts/*.
My_personal_ssh	:	SSH parameters. Refer to your computer core facility
My_project_title	:	This is the name of the folder of your project on the remote server [automatically generated by RNApipCreateNewProject].
Reference_genome	:	The genome your data will be aligned to. Make sure your core facility has this genome reference installed on your cluster and that the extensions of the files are '.fa'.
Local_path_to_proj	:	[Automatically generated by RNApipCreateNewProject].
Local_path_to_NEAT	:	[Automatically generated by RNApipCreateNewProject].
Proj_TaxonDatabase	:	See NEAT for RNAseq.
Proj_TaxonDatabaseDic	:	See NEAT for RNAseq.
Remote_path_to_proj	:	Full path to your project folder (without the project name) [automatically generated].
Remote_path_to_NEAT	:	Full path to your NEAT folder. Note that in our example, we have created our project within the RNApip folder itself, but users can freely decide to create a dedicated folder for all of their RNApip projects.
Remote_path_to_orifastq.gz		Full path to where your compressed .fastq.gz files are. Usually, your sequencing core facility will let you know where they store these files. Note that all .fastq.gz files can be kept in a single location, they do not need to be copied to your folder.
Remote_path_to_chrLens.dat		Path to a *.dat* file containing chromosome information for your reference genome. Refer to your computer core facility.
Remote_path_to_RefGen.fasta		Path to folder containing your reference genome files. This folder needs to contain the Indexes (**.ebwt* files) as well as the fasta file (*.fa*). Refer to your computer core facility.
Aligner_algo_short	:	Tophat, a splice-aware aligner, will be used by default for RNAseq experiments. If other algorithms are used, modify the *AdvancedSettings.txt* file accordingly.
Paired_end_seq_run	:	"0" for single end sequencing. "1" for paired end sequencing.
Steps_to_execute_pipe	:	Users can choose from the following tasks:<br>• *Unzip*<br>• *Qc*<br>• *Map*<br>• *Filter*<br>• *Cleanfolders*<br>• *GRanges*<br>If you do not want to run all of these, simply delete them for the

		Targets.txt or rename them. Once ran, RNApip will change the value of these from '*unzip*' to '*unzip_DONE*'. Obviously, a certain hierarchy has to be followed, e.g. attempting to filter reads without having previously mapped them (in the same run or in a previous run) will not work. Note that '*qc*' requires Thomas Girke's systemPipeR package; map requires bwa; the default *peacalling* requires the R package SPP; *filter* requires samtools. Refer below for exact requirements.
Sample naming	:	For single-end (SE) runs, fill in the '*SAMPLES INFO*' section. Characters including spaces, dollar signs and other well-known special characters should not be used. Underscores should be the reference character to delimit words. For paired-end sequencing runs, in addition to filling in the first section, fill in the '*PE CORRESPONDING SECTION*', which correspond to the .fastq files containing the reads from the reverse strand. The names of these files need to be identical to the exception of adding a '*_R2*' at the end of the name. For example, if the first file name is '*PSa29-5_noDox*, the corresponding reverse strand file will be named '*PSa29-5_noDox_R2*'. The order in which the files appear also needs to be identical in both sections. Please refer to the example Targets.txt file for more information.

Please modify the *Targets.txt* file according to your needs. To modify the Targets.txt file, we suggest users get accustomed to using plain text editors such as TextWrangler as it will avoid including spaces and special characters. In addition, it is worth making sure that the parameters in the *AdvancedSettings.txt* files are correct, especially the unzip command and extension as well as the alignment command (Tophat).

The paths to the reference genomes should be obtained from your computer core facility (system administrator), as they are the ones usually maintaining these up to date. Note here that the reference genome files should have an '*.fa*' extension (e.g. mm9.fa). Please make sure that your core has named these files accordingly as any other extension will lead the pipeline to abort prematurely.

Note here that *#Remote_path_to_RefGen.fasta* refers to the path to the folder in which '*.fa*' files are located, not to the file itself. This is different than in ChIPpip.

To avoid repeating these steps at each new NEAT (RNApip) project creation, we suggest you modify the *original Targets.txt* file that is used as template when creating a new ChIPpip project. This file can be found in *~/NEAT/RNApip/scripts/NewRNApipProject/DataStructure/Targets.txt*.

3.3 Running NEAT Part 2

Once the *Targets.txt* file is correctly set up, users can run the *2_Run_NEAT.app*.

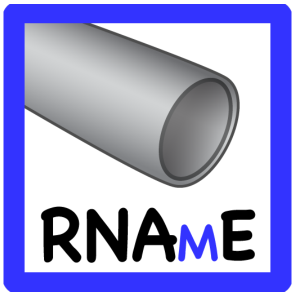

This script will execute the tasks specified in the *Targets.txt* file.

After asking the user to enter his/her ssh password (twice), this will launch the pipeline and will prompt a summary of the user's parameters. NEAT automatically manages all creations and batch submissions of jobs, dependencies, ordering of files, queuing, etc. If the cluster is using TORQUE, the processes can be followed on the terminal using the ***qstat (or bjobs)*** command (type ***qstat (or bjobs)*** in your terminal) after ssh-ing into your remote cluster. To ssh into a remote cluster, type in the following commands into a terminal window replacing your username and server address:

[MY_COMPUTE~]$ ***ssh username@serveradress.edu***
password

```
[username@setrveradress ~]$
```

Type ***qstat (or bjobs)*** to follow the status of the pipeline. Briefly, *Q* stands for queuing, *R* for running, *E* for exiting and *H* for holding.

Once the pipeline has finished, it will notify users of its status by email (if applicable).

The mock data provided as a test example should take no more than one hour to run, usually a lot less.

4 NEAT part 3 and 4

4.1 Running NEAT Part 3

4.1.1 Step 1: Download a RNApip project

To transfer a NEAT project from a remote server to a local computer, double click the RNAmE 3_*Transfer.app*.

Users will be prompted to locate the NEAT directory and where they want to save their RNApip project. In this example, the NEAT directory is on the desktop.

The *3_Transfer.app* will use all the information found in your Targets file to download the RNApip project from the remote server to your local computer. Please be attentive as users will need to enter their ssh password several times.

Downloading an entire project should not take more than a few minutes.

4.2 Running NEAT Part 4

4.2.1 Step 2: Run a RNAmE analysis

Once the project has been downloaded, users can run the proper RNAmE analysis. To this end, double click the NEAT *4_Analyze.app*.

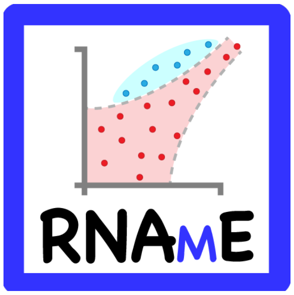

Users will be asked to locate the NEAT folder (also saved on the desktop in this example). Users will also need to choose a mart object. Finally, users will be asked to locate the RNApip folder (the one just downloaded). In this example, the RNApip project was downloaded to the desktop. Running the analysis using the test data should take less than a minute.

4.3 Outputs

4.3.1 Logs

Each time RNAmE is run, a log file is created and named using the date and time. This file is save in the *~/MY_NEW_RNA_PROJECT/logs/* directory. We strongly encourage users to initially look at these files, as any error that might have occurred will be saved there. Usually, if no error is prompted from the terminal, RNAmE has terminated correctly. Also, please note that if there are unrecognized chromosome names such as random chromosomes, warning messages will appear. In the test data, there are 50 or more warnings. These can usually be disregarded.

4.3.2 Count tables and RPKM tables

RNAmE will generate count tables that should be self-explanatory. In brief, rows correspond to genes and columns correspond to samples.

4.3.3 Differentially expressed gene plots

RNAmE generates pdf plots that are saved in *~/MY_NEW_RNA_PROJECT/plots/*. General smear plots are created as a quick EDA between samples and replicates. In additional to smear plots, RNAmE generates all possible combinations of QQplots, with highlighted up and downregulated genes. More information on these genes can be found in the corresponding count tables. Finally, RNAmE generates gene lists meant to be fed to Gorilla, a well-established GO term EDA software. Venn diagrams are produced if selected. Users should note that computational time increases exponentially with the number of datasets as all combinations of Venn plots are created.

The test dataset generated the below presented smear plots. Obviously, the limited number of reads in the test data makes this plot relatively uninformative, but analyzing your own data will give much better resolution.

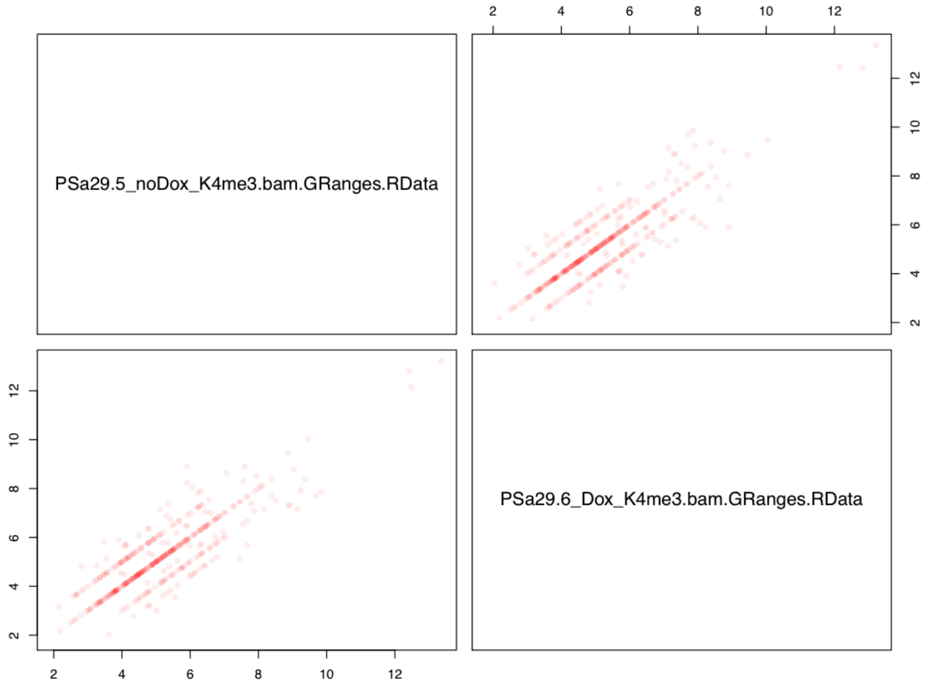

4.4 Advanced settings

4.4.1 Bam files and GRanges

Bam files are generated during the initial phase (step 2) but are not automatically downloaded to the local computer. Rather, the GRanges files, which are often two orders of magnitude smaller in size (respectively several Gb vs tens of Mb) are dowlonaded. If required, bam files are always stored in the 'bam' folder on the remote server.

4.4.2 Consolidating projects

Consolidating projects is very easy. Users who intend to do so will simply need to change the *Targets.txt* file and copy-paste the Granges objects (or bam files) from one folder to the other. Other files and folder can be left as is.

5 Version information and required packages

Version: 0.5.9-r16

Program: samtools (Tools for alignments in the SAM format)
Version: 0.1.18 (r982:295)

R version 3.1.0 (2014-04-10)
Platform: x86_64-redhat-linux-gnu (64-bit)

R packages (with dependencies) required to run RNApip:

- systemPipeR (systemPipeR_0.99.0)
- ...

R packages (with dependencies) required to run RNAmE:

- Rsamtools
- GenomicRanges
- GenomicAlignments
- caTools
- VennDiagram

sessionInfo()

R version 3.1.2 (2014-10-31)

Platform: x86_64-apple-darwin10.8.0 (64-bit)

locale:

[1] en_US.UTF-8/en_US.UTF-8/en_US.UTF-8/C/en_US.UTF-8/en_US.UTF-8

attached base packages:

[1] grid parallel stats4 stats graphics grDevices utils

[8] datasets methods base

other attached packages:

[1] VennDiagram_1.6.9 caTools_1.17.1 GenomicAlignments_1.2.1

[4] Rsamtools_1.18.2 Biostrings_2.34.1 XVector_0.6.0

[7] GenomicRanges_1.18.3 GenomeInfoDb_1.2.3 IRanges_2.0.1
[10] S4Vectors_0.4.0 BiocGenerics_0.12.1

loaded via a namespace (and not attached):
[1] base64enc_0.1-2 BatchJobs_1.5 BBmisc_1.8 BiocParallel_1.0.0
[5] bitops_1.0-6 brew_1.0-6 checkmate_1.5.0 codetools_0.2-9
[9] DBI_0.3.1 digest_0.6.4 fail_1.2 foreach_1.4.2
[13] iterators_1.0.7 RSQLite_1.0.0 sendmailR_1.2-1 stringr_0.6.2
[17] tools_3.1.2 zlibbioc_1.12.0

6 Reported bugs

Refer to the complete guide for reported bugs

7 Funding

This pipeline was developed in part with funding from the Swiss National Science Foundation P300P3_158516 and in part through the R37 GM48405-21 grant awarded to Robert E Kingston

8 References

- Kharchenko P, Tolstorukov M & Park P. (2008) Design and analysis of ChIP-seq experiments for DNA-binding proteins. *Nature Biotechnology* **26**, 1351 - 1359
- Girke T. (2014) systemPipeR: NGS workflow and report generation environment. URL https://github. com/tgirke/systemPipeR.
- Li H. and Durbin R. (2009) Fast and accurate short read alignment with Burrows-Wheeler Transform. *Bioinformatics*, 25:1754-60.
- Michael Lawrence, Huber W, Pagès H, Aboyoun P, Carlson M, Gentleman R, Morgan MT, and Carey VJ. (2013) Software for computing and annotating genomic ranges. *PLoS Comput. Biol.*, 9(8):e1003118.
- Li H and Durbin R. (2009) Fast and accurate short read alignment with Burrows-Wheeler transform. *Bioinformatics*, 25(14): 1754–1760.
- Li H. (2013) Aligning sequence reads, clone sequences and assembly contigs with BWA-MEM. URL http: //arxiv.org/abs/1303.3997.
