# Supplementary material for: NEAT: a framework for building fully automated NGS pipelines and analyses
Source: BMC Bioinformatics. 2016 Feb 1;17:53. doi: 10.1186/s12859-016-0902-3 (PMC4736651; doi:10.1186/s12859-016-0902-3)
Supplement: Additional file 3: — Code architecture. Architecture of the provided NEAT ChIPseq project after completion of all steps on the remote server. The color code highlights which files are created during which step as well as where they are stored. (PDF 34 kb) [file 12859_2016_902_MOESM3_ESM.pdf]

Arborescence of a NEAT project

(based on ChIPseq project ran on Torque manager)

After unzip (or from start)
After QC
After ChIPRX
After map
After filter
After peakcalling
After cleanfiles
After GRanges

```
|- aligned
          |------ *
                    `-------------- *.bai
                    `-------------- *.bam
                    `-------------- *.sam
                    `-------------- *.sai
                    `-------------- *.u.sam
                    `-------------- *.u.sortedwpcr.bam
                    `-------------- *.u.unsorted.bam
|- bam
          `------ *.bai
          `------ *.bam
|- bam_RX
          `------ *_RX.bai
          `------ *_RX.bam
|- DataStructure
          `------ AdvancedSettings.txt
          |------ mm9
          `------ statTable.bed
          `------ Targets.txt
|- fastq
          `------ *.fastq
|- GRangesRData
          `------ *.bam.GRanges.RData
|- GRangesRData_RX
          `------ *_RX.bam.GRanges.RData
|- peakcalling
          |------ bigwig
          |------ broadPeak
          |------ narrowPeak
          |------ *
                    `-------------- *.binding.positions.txt
                    `-------------- *.broadPeak
                    `-------------- *.crosscorrelation.pdf
                    `-------------- *.denstiy.wig
                    `-------------- *.narrowPeak
|- QC
          `------ QCReport.pdf

|- scripts
          |------ cleanfiles
                    `-------------- cleanfiles.sh
                    `-------------- *_cleanfiles.sh
                    |-------- qsub
          |------ filter
                    `-------------- cleanfiles.sh
                    `-------------- *_cleanfiles.sh
                    |-------- qsub
          |------ granges
                    `-------------- granges_qsub.sh
                    `-------------- granges.sh
                    |-------- qsub
          |------ iterate
                    `-------------- ChIPpip_ProjectName.pl
                    `-------------- ChIPpip_ProjectName.sh
                    `-------------- Iterate_ProjectName.sh
                    |-------- qsub
          |------ map
                    `-------------- map.sh
                    `-------------- *_map.sh
                    |-------- qsub
          |------ peakcalling
                    `-------------- peakcalling.sh
                    `-------------- *_peakcalling.sh
                    |-------- qsub
          |------ QC
                    `-------------- QC_qsub.sh
                    `-------------- QC.sh
                    |-------- qsub
|- wig
          `------ *.bam.GRanges.RData.wig
```
