# Supplementary figures and images for: NEAT: a framework for building fully automated NGS pipelines and analyses
Source: BMC Bioinformatics. 2016 Feb 1;17:53. doi: 10.1186/s12859-016-0902-3 (PMC4736651; doi:10.1186/s12859-016-0902-3)

# RNaseq

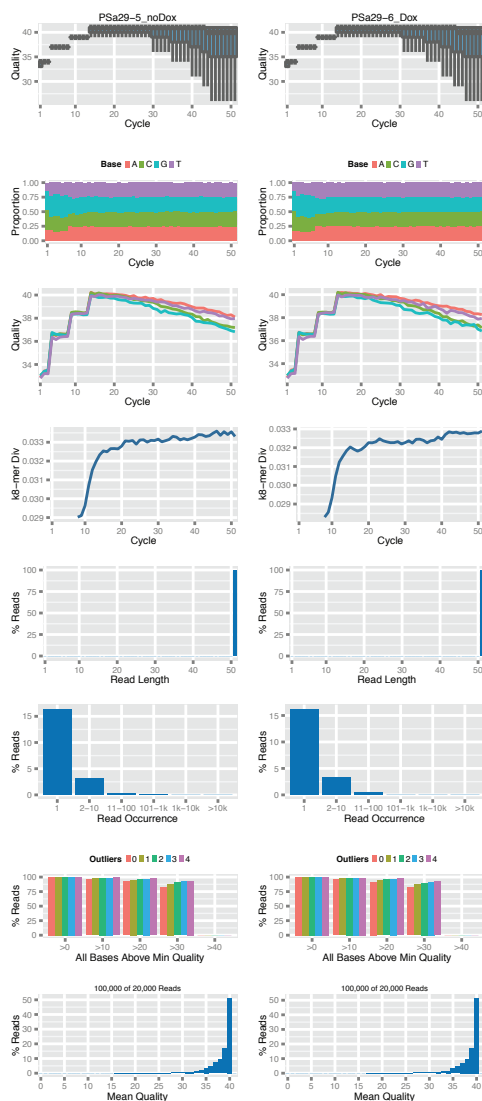

# ChIPseq

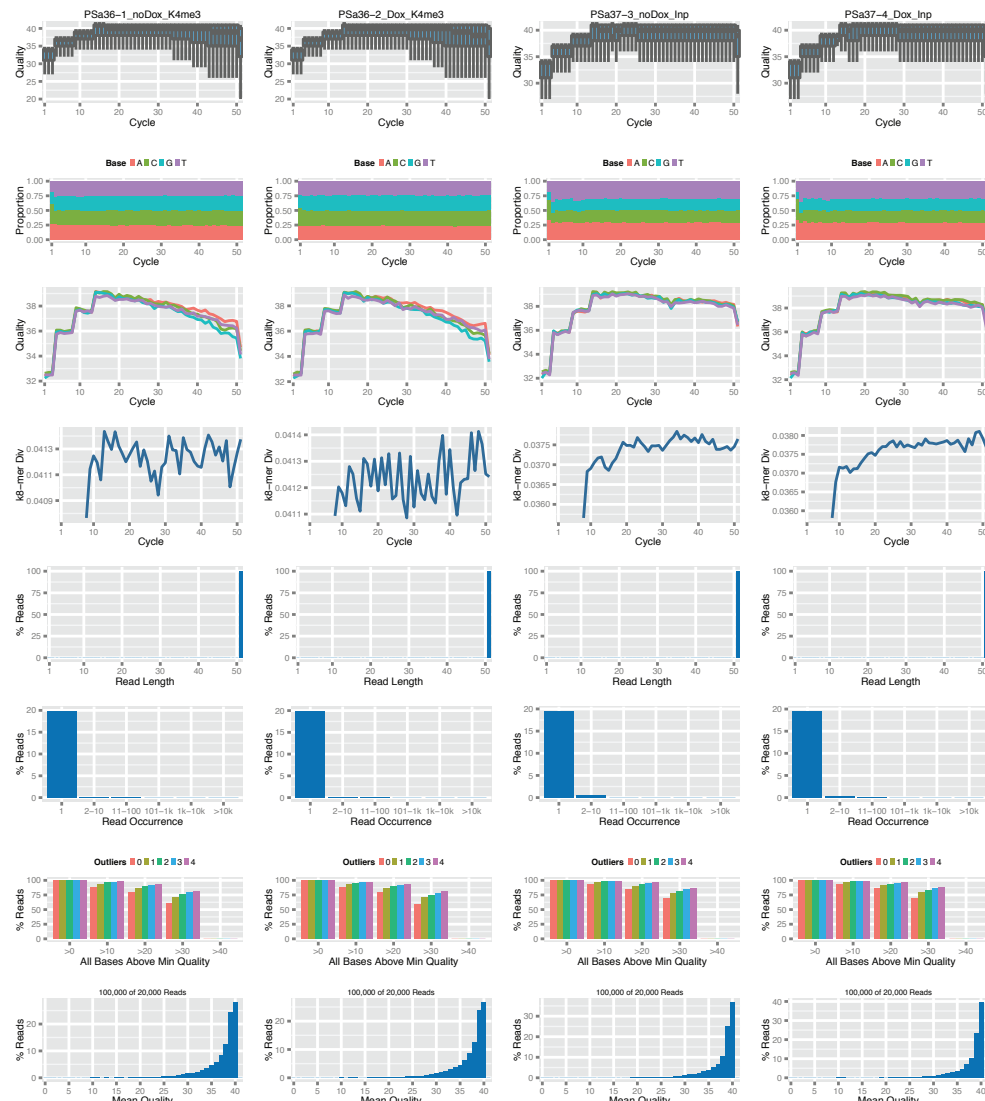

Supplement: Additional file 4: — QC report. QC report generated by NEAT when running the ChIP- and RNAseq test data set. (PDF 889 kb) [file 12859_2016_902_MOESM4_ESM.pdf]
