# Supplementary material for: NEAT: a framework for building fully automated NGS pipelines and analyses
Source: BMC Bioinformatics. 2016 Feb 1;17:53. doi: 10.1186/s12859-016-0902-3 (PMC4736651; doi:10.1186/s12859-016-0902-3)
Supplement: Additional file 5: — Quick guide to add custom modules. Step-by-step guide for the addition of custom modules. (PDF 360 kb) [file 12859_2016_902_MOESM5_ESM.pdf]

NEAT

Adding modules

Patrick Schorderet

Patrick.schorderet@molbio.mgh.harvard.edu

June 2015

1 INTRODUCTION 4

2 ARCHITECTURE 5

2.1 GENERAL ARCHITECTURE 5

2.2 CODE ARCHITECTURE 6

1 Introduction

NExt generation ***A***nalysis ***T***oolbox (NEAT) is a perl/R package that supports users during the analysis of next generation sequencing (NGS).

NEAT is versatile and easy to modify. In this tutorial, we will show how to add a custom module to NEAT. Adding a new module has been made as easy as possible by automating all the repetitive tasks such as job creation, batch submission and queuing. Adding a new module usually falls down to a single line of code.

2 Architecture

2.1 General architecture

NEAT contains different modules (yellow boxes) than can be modified and/or added.

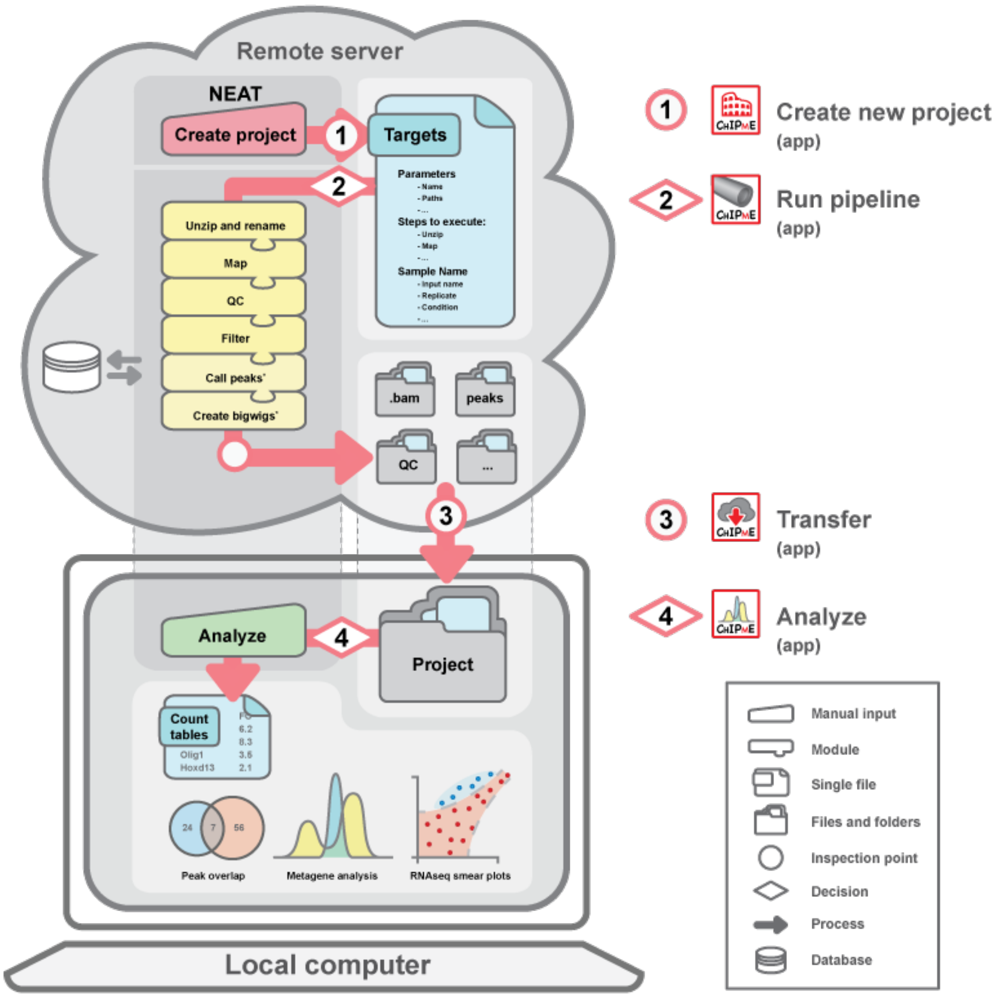

Fig.1 NEAT architecture. NGS data can be analyzed using NEAT in less than a day. Users follow a logical 4-step process, including the creation of a new project, running the pipeline on a remote server or in the cloud, transferring the data to a local computer and proceeding to the analysis.

2.2 Code architecture

The main code of NEAT (in the example of ChIPseq projects) is found in *./NEAT/ChIPpip/scripts/ChIPpip.pl*. The code is well annotated, highly redundant and shuld be self-explanatory to advanced users. Main modules are easily identifiable and customizable. A brief summary of how each module is built is depicted below.

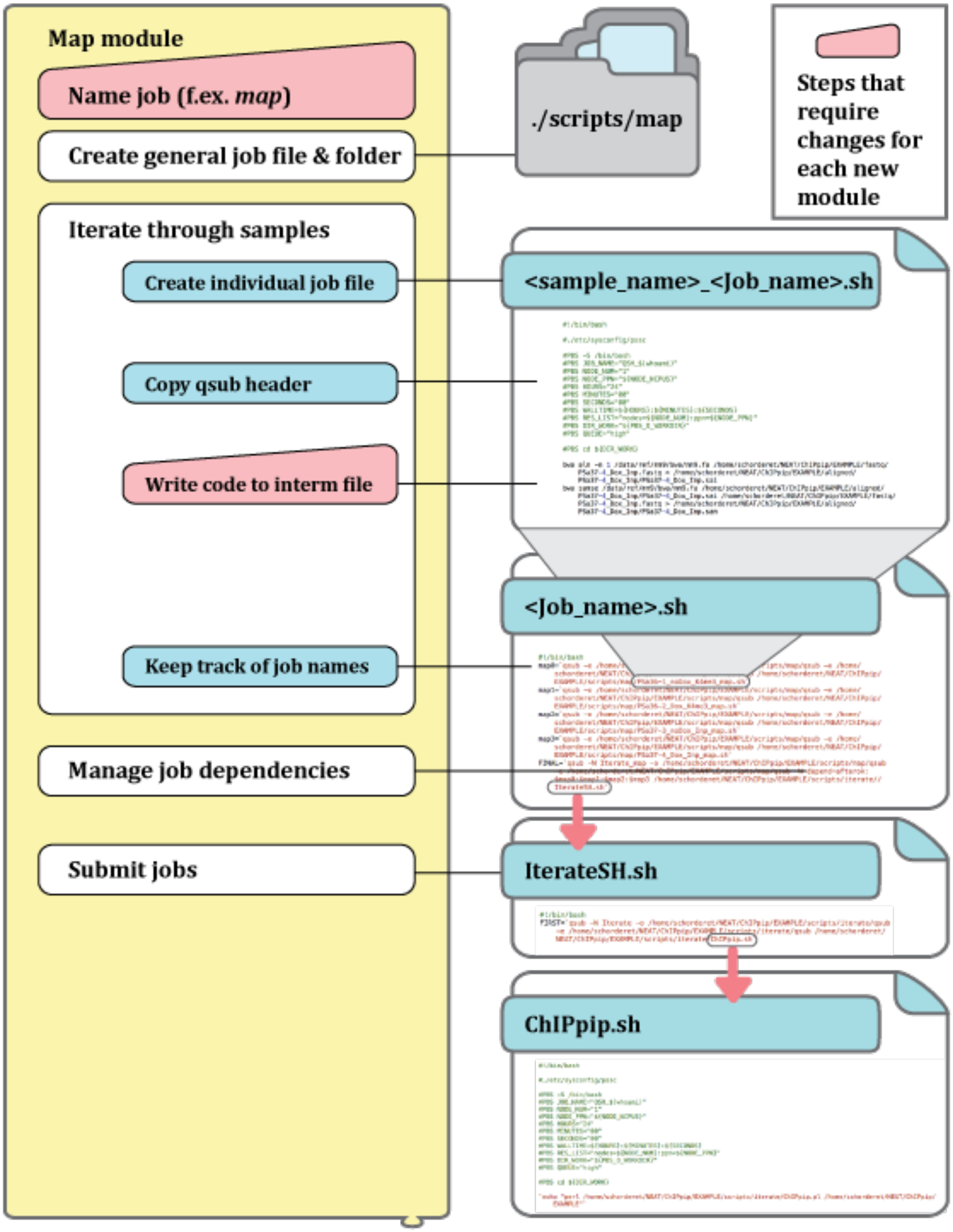

Map module
Name job (f.ex. map)
Create general job file & folder
Iterate through samples
Create individual job file
Copy qsub header
Write code to interm file
Keep track of job names
Manage job dependencies
Submit jobs
./scripts/map
Steps that require changes for each new module
<sample_name>_<Job_name>.sh
<Job_name>.sh
IterateSH.sh
ChIPpip.sh

Map module

```
#*------------------------------------------------------------*
# Mapping sequences with bwa

if( $map =~ "TRUE" ){

    print "\n\n*^*^*^*^*^*^*^*^*^*^*^*^*^*^*^*^*^*^*^*^*\n";
    print "\n Mapping fastq files\n";

    my $iterateJobName = "Iterate_<YOUR_JOB_NAME_HERE>";
    my $myJobName            = "<YOUR_JOB_NAME_HERE>";
    my $path2qsub            = "$tmpscr/$myJobName/qsub";

    # Create file to store jobs in
    unless( -d "$tmpscr/$myJobName" )        { `mkdir $tmpscr/$myJobName`; }
    unless( -d "$path2qsub" )                { `mkdir $path2qsub`; }
    my $QSUB     = "$tmpscr/$myJobName/$myJobName\.sh";
    open $QSUB, ">", "$QSUB" or die "Can't open '$QSUB'";
    print $QSUB "#!/bin/bash\n";
    close $QSUB;
    `chmod 777 $QSUB`;
    print "\n Store all of the following '$myJobName' jobs in $QSUB \n";
    my @myJobs;


    foreach my $i (0 .. $#samplesInputs) {

        # Prepare a personal qsub script
        my $QSUBint          = "$tmpscr/$myJobName/$samplesInputs[$i]\_$myJobName\.sh";
        `cp $scrhead $QSUBint`;

        # Create a directory
        my $path2currentSampleDir    = "$path2aligned/$samplesInputs[$i]";
        unless( -d "$path2currentSampleDir" )    { `mkdir $path2currentSampleDir`; }

        #-+-+-+-+-+-+-+-+-+-+-+-+-+-+-+-+-+-+-+-+-+-+-+-+-+-+-+-+-+-+-+-+-+-+-+-+-+
        #-+-+-+-+-+-+-+-+-+-+-+-+-+    IMPORTANT CODE HERE    -+-+-+-+-+-+-+-+-+-+-+-+-+
        my $cmd              = "<YOUR_CUSTOM_COMMAND_HERE>";
        `echo "$cmd" >> $QSUBint`;
        #-++--++--++--++--++--++--++--++--++--++--++--++--++--++--++--++--++--++--++--


        #------------------------------------
        # Keep track of the jobs in @myJobs
        my $jobName     = "$myJobName$i";
        push(@myJobs, $jobName);
        $cmd                 = "$jobName=`qsub -o $path2qsub -e $path2qsub $QSUBint`";
        open $QSUB, ">>", "$QSUB" or die "Can't open '$QSUB'";
        print $QSUB "$cmd\n";
        close $QSUB;

    }

    #*------------------------------------------------------------*
    # Change Targets.txt file for next iteration
    print "\n------------------------------------------------------------------------\n";
    print "\n Changing '$myJobName' variable to FALSE and proceed";
    `/usr/bin/perl -p -i -e "s/$myJobName/$myJobName\_DONE/gi" $Targets`;

    #*------------------------------------------------------------*
    # Prepar file containing the jobs to run

    # Add the next job line to the $mapQSUB
    foreach( @myJobs ){ $_ = "\$".$_ ; }
    my $myJobsVec        = join(":", @myJobs);
    my $finalcmd         = "FINAL=\`qsub -N $iterateJobName -o $path2qsub -e $path2qsub -W depend=afterok\:$myJobsVec $iterateSH\`";
    open $QSUB, ">>", "$QSUB" or die "Can't open '$QSUB'";
    print $QSUB "$finalcmd\n";
    close $QSUB;

    #*------------------------------------------------------------*
    # Submit jobs to run

    print "\n\n========================================================================\n";
    print "\n Submitting job to cluster: \t `sh $QSUB` \n";
    `sh $QSUB`;

    #*------------------------------------------------------------*
    # Exit script

    print "\n~~~~~~~~~~~~~~~~~~~~~~~~~~~~~~~~~~~~~~~~~~~~~~~~~~~~~~~~~~~~~~~~~~~~~~~~\n";
    print "\n Exiting $myJobName section with no known error \n";
    print "\n~~~~~~~~~~~~~~~~~~~~~~~~~~~~~~~~~~~~~~~~~~~~~~~~~~~~~~~~~~~~~~~~~~~~~~~~\n\n\n";

    exit 0;

}
```

If job is TRUE, enter module (modify the 'Steps_to_execute variables at the top of the code)

Name your job (f.ex map)

A file is created with YOUR_JOB_NAME. This file will countain the flow of each individual-job related to this job.

For loop that iterates through all samples and inputs. To iterate through samples only, use the @samples table instead of the @samplesInputs table.

For each individual job, create a qsub-like file (in which the job code will be) by copying the qsub header ($scrhead) into the intermediary $QSUBint file

YOUR CUSTOMIZE CODE GOES HERE

Keep track of job names to ensure job dependencies (see below)

Modify the Targets.txt file to mark the job as '_DONE'

Prepare code for the next iteration ($IterateSH). This iteration depends on the completion of all the current jobs ($myJobsVec)

Submit job to cluster and exit

To add a module, copy-paste an entire block and change the following:

- Name of job ($jobName)
- Make sure the loop is correctly set (looping over all samples, inputs, etc)
- Add your code line under the “important code here” section
- Lastly, add the variable to the others in the “# Steps_to_execute_pipe” section at the top portion of the code.
