# Supplementary material for: NEAT: a framework for building fully automated NGS pipelines and analyses
Source: BMC Bioinformatics. 2016 Feb 1;17:53. doi: 10.1186/s12859-016-0902-3 (PMC4736651; doi:10.1186/s12859-016-0902-3)
Supplement: Additional file 6: — Code architecture schematic. Example of a module’s architecture in NEAT. The left part schematically represents the different steps that constitute each module. The right part represents some example code and how it is imbricated. The example reflects NEAT run on a torque manager system (qsub/PBS) though NEAT can be run on other systems as well including LSF clsuters (bsub). (PDF 960 kb) [file 12859_2016_902_MOESM6_ESM.pdf]

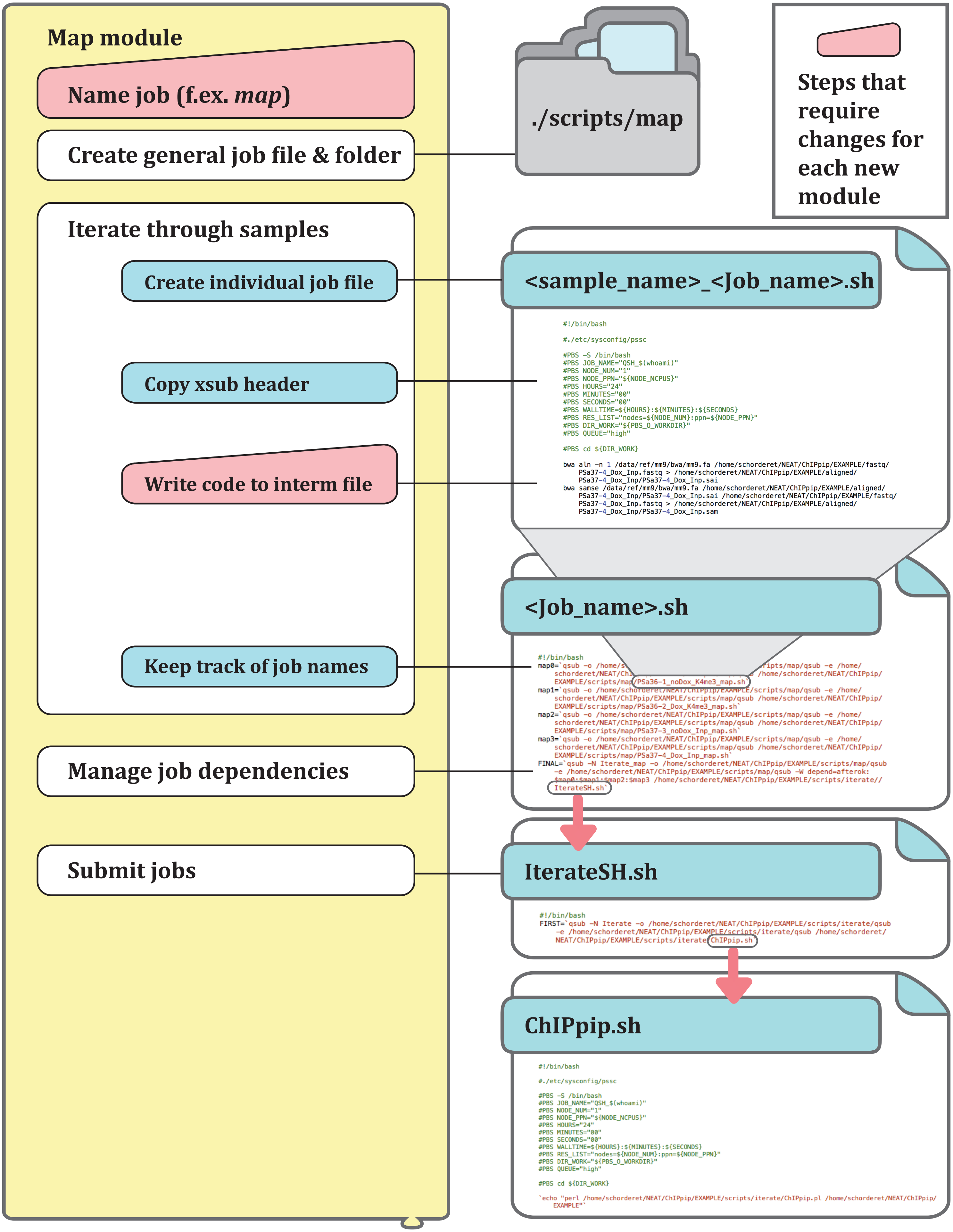

Map module
Name job (f.ex. map)
Create general job file & folder
Iterate through samples
Create individual job file
Copy xsub header
Write code to interm file
Keep track of job names
Manage job dependencies
Submit jobs
./scripts/map
Steps that require changes for each new module
<sample_name>_<Job_name>.sh
#!/bin/bash
#./etc/sysconfig/pssc
#PBS -S /bin/bash
#PBS JOB_NAME="QSH_$(whoami)"
#PBS NODE_NUM="1"
#PBS NODE_PPN="${NODE_NCPUS}"
#PBS HOURS="24"
#PBS MINUTES="00"
#PBS SECONDS="00"
#PBS WALLTIME=${HOURS}:${MINUTES}:${SECONDS}
#PBS RES_LIST="nodes=${NODE_NUM}:ppn=${NODE_PPN}"
#PBS DIR_WORK="${PBS_O_WORKDIR}"
#PBS QUEUE="high"
#PBS cd ${DIR_WORK}
bwa aln -n 1 /data/ref/mm9/bwa/mm9.fa /home/schorderet/NEAT/ChIPpip/EXAMPLE/fastq/
PSa37-4_Dox_Inp.fastq > /home/schorderet/NEAT/ChIPpip/EXAMPLE/aligned/
PSa37-4_Dox_Inp/PSa37-4_Dox_Inp.sai
bwa samse /data/ref/mm9/bwa/mm9.fa /home/schorderet/NEAT/ChIPpip/EXAMPLE/aligned/
PSa37-4_Dox_Inp/PSa37-4_Dox_Inp.sai /home/schorderet/NEAT/ChIPpip/EXAMPLE/fastq/
PSa37-4_Dox_Inp.fastq > /home/schorderet/NEAT/ChIPpip/EXAMPLE/aligned/
PSa37-4_Dox_Inp/PSa37-4_Dox_Inp.sam
<Job_name>.sh
#!/bin/bash
map0=`qsub -o /home/s
ripts/map/qsub -e /home/
schorderet/NEAT/Chi
/home/schorderet/NEAT/ChIPpip/
EXAMPLE/scripts/map/PSa36-1_noDox_K4me3_map.sh`
map1=`qsub -o /home/schorderet/NEAT/ChIPpip/EXAMPLE/scripts/map/qsub -e /home/
schorderet/NEAT/ChIPpip/EXAMPLE/scripts/map/qsub /home/schorderet/NEAT/ChIPpip/
EXAMPLE/scripts/map/PSa36-2_Dox_K4me3_map.sh`
map2=`qsub -o /home/schorderet/NEAT/ChIPpip/EXAMPLE/scripts/map/qsub -e /home/
schorderet/NEAT/ChIPpip/EXAMPLE/scripts/map/qsub /home/schorderet/NEAT/ChIPpip/
EXAMPLE/scripts/map/PSa37-3_noDox_Inp_map.sh`
map3=`qsub -o /home/schorderet/NEAT/ChIPpip/EXAMPLE/scripts/map/qsub -e /home/
schorderet/NEAT/ChIPpip/EXAMPLE/scripts/map/qsub /home/schorderet/NEAT/ChIPpip/
EXAMPLE/scripts/map/PSa37-4_Dox_Inp_map.sh`
FINAL=`qsub -N Iterate_map -o /home/schorderet/NEAT/ChIPpip/EXAMPLE/scripts/map/qsub
-e /home/schorderet/NEAT/ChIPpip/EXAMPLE/scripts/map/qsub -W depend=afterok:
$map0:$map1:$map2:$map3 /home/schorderet/NEAT/ChIPpip/EXAMPLE/scripts/iterate//
IterateSH.sh`
IterateSH.sh
#!/bin/bash
FIRST=`qsub -N Iterate -o /home/schorderet/NEAT/ChIPpip/EXAMPLE/scripts/iterate/qsub
-e /home/schorderet/NEAT/ChIPpip/EXAMPLE/scripts/iterate/qsub /home/schorderet/
NEAT/ChIPpip/EXAMPLE/scripts/iterate/ChIPpip.sh`
ChIPpip.sh
#!/bin/bash
#./etc/sysconfig/pssc
#PBS -S /bin/bash
#PBS JOB_NAME="QSH_$(whoami)"
#PBS NODE_NUM="1"
#PBS NODE_PPN="${NODE_NCPUS}"
#PBS HOURS="24"
#PBS MINUTES="00"
#PBS SECONDS="00"
#PBS WALLTIME=${HOURS}:${MINUTES}:${SECONDS}
#PBS RES_LIST="nodes=${NODE_NUM}:ppn=${NODE_PPN}"
#PBS DIR_WORK="${PBS_O_WORKDIR}"
#PBS QUEUE="high"
#PBS cd ${DIR_WORK}
`echo "perl /home/schorderet/NEAT/ChIPpip/EXAMPLE/scripts/iterate/ChIPpip.pl /home/schorderet/NEAT/ChIPpip/
EXAMPLE"`
