# Supplementary material for: NEAT: a framework for building fully automated NGS pipelines and analyses
Source: BMC Bioinformatics. 2016 Feb 1;17:53. doi: 10.1186/s12859-016-0902-3 (PMC4736651; doi:10.1186/s12859-016-0902-3)
Supplement: Additional file 7: — Code architecture. Code architecture for the additional of custom modules. Custom code should replace the red font (make sure the loop is correct depending on whether it is a ChIPseq or RNAseq module). The module backbone as well as the submission procedures are robust, highly repetitive and will automatically manage job submission and queuing. (PDF 458 kb) [file 12859_2016_902_MOESM7_ESM.pdf]

Map module

```
#*---------------------------------------------------------------------*
# Mapping sequences with bwa

if( $map =~ "TRUE" ){

        print "\n\n*-*-*-*-*-*-*-*-*-*-*-*-*-*-*-*-*-*-*-*-*-*-\n";
        print "\n Mapping fastq files\n";

        my $iterateJobName          = "Iterate_<YOUR_JOB_NAME_HERE>";
        my $myJobName               = "<YOUR_JOB_NAME_HERE>";
        my $path2qsub               = "$tmpscr/$myJobName/$SUBkey";

        # Create file to store jobs in
        unless( -d "$tmpscr/$myJobName" )      { `mkdir $tmpscr/$myJobName`; }
        unless( -d "$path2qsub" )              { `mkdir $path2qsub`; }
        my $QSUB       = "$tmpscr/$myJobName/$myJobName\.sh";
        open $QSUB, ">", "$QSUB" or die "Can't open '$QSUB'";
        print $QSUB "#!/bin/bash\n";
        close $QSUB;
        `chmod 777 $QSUB`;
        print "\n Store all of the following '$myJobName' jobs in $QSUB \n";
        my @myJobs;


        foreach my $i (0 .. $#samplesInputs) {

                # Prepare a personal qsub script
                my $QSUBint            = "$tmpscr/$myJobName/$samplesInputs[$i]\_$myJobName\.sh";
                `cp $scrhead $QSUBint`;

                # Create a directory
                my $path2currentSampleDir     = "$path2aligned/$samplesInputs[$i]";
                unless( -d "$path2currentSampleDir" )     { `mkdir $path2currentSampleDir`; }


                #-+-+-+-+-+-+-+-+-+-+-+-+-+-+-+-+-+-+-+-+-+-+-+-+-+-+-+-+-+-+-+-+-+-+-+-+-+-+-+-+-+-+-+-+-+-+-+-+-+-+-+-+-+-+-+
                #-+-+-+-+-+-+-+-+-+-+-+-+-+-+-+-+-+-+-+-+-+        IMPORTANT CODE HERE        -+-+-+-+-+-+-+-+-+-+-+-+-+-+-+-+-+-+-+-+-+-+-+
                my $cmd                = "<YOUR_CUSTOM_COMMAND_HERE>";
                `echo "$cmd" >> $QSUBint`;
                #--++--++--++--++--++--++--++--++--++--++--++--++--++--++--++--++--++--++--++--++--++--++--++--++--++--++--++--

                #---------------------------------------------
                # Keep track of the jobs in @myJobs
                my $jobName     = "$myJobName$i";
                push(@myJobs, $jobName);
                if($SUBkey =~ "qsub"){  $cmd   = "$jobName=`$SUBcommand -o $path2qsub -e $path2qsub $QSUBint`";}
                if($SUBkey =~ "bsub"){  $cmd    = "$SUBcommand -J $jobName -o $path2qsub\/$jobName.out -e $path2qsub\/$jobName.err $QSUBint";}
                open $QSUB, ">>", "$QSUB" or die "Can't open '$QSUB'";
                print $QSUB "$cmd\n";
                close $QSUB;

        }

        #*---------------------------------------------------------------------*
        # Change Targets.txt file for next iteration
        print "\n-----------------------------------------------------------------------------------------------------\n";
        print "\n Changing '$myJobName' variable to FALSE and proceed";
        `/usr/bin/perl -p -i -e "s/$myJobName/$myJobName\_DONE/gi" $Targets`;

        #*---------------------------------------------------------------------*
        # Prepar file containing the jobs to run

        # Add the next job line to the $mapQSUB
        foreach( @myJobs ){ $_ = "\$".$_ ; }
        my $myJobsVec          = join(":", @myJobs);

        if($SUBkey =~ "qsub"){
                foreach( @myJobs ){ $_ = "\$".$_ ; }
                my $myJobsVec   = join(":", @myJobs);
                $finalcmd = "FINAL=\`$SUBcommand -N $iterateJobName -o $path2qsub -e $path2qsub $IterateSH`";
        }
         if($SUBkey =~ "bsub"){
                foreach( @myJobs ){ $_ = "$SUBdependCondition\(\"".$_."\")" ; }
                my $myJobsVec   = join(" && ", @myJobs);
                $finalcmd      = "$SUBcommand -J $iterateJobName -o $path2qsub\/$iterateJobName.out -e $path2qsub\/$iterateJobName.err $IterateSH";
        }

        open $QSUB, ">>", "$QSUB" or die "Can't open '$QSUB'";
        print $QSUB "$finalcmd\n";
        close $QSUB;

        #*---------------------------------------------------------------------*
        # Submit jobs to run
        `sh $QSUB`;

        #*---------------------------------------------------------------------*
        # Exit script
        print "\n~~~~~~~~~~~~~~~~~~~~~~~~~~~~~~~~~~~~~~~~~~~~~~~~~~~~~~~~~~~~~~~~~~~~~~~~~~~~~\n";
        print "\n Exiting $myJobName section with no known error \n";
        print "\n~~~~~~~~~~~~~~~~~~~~~~~~~~~~~~~~~~~~~~~~~~~~~~~~~~~~~~~~~~~~~~~~~~~~~~~~~~~\n\n\n";

        exit 0;
}
```

If job is TRUE, enter module (modify the 'Steps_to_execute variables at the top of the code)

Name your job (f.ex map)

A file is created with YOUR_JOB_NAME. This file will countain the flow of each individual-job related to this job.

For loop that iterates through all samples and inputs. To iterate through samples only, use the @samples table instead of the @samplesInputs table.

For each individual job, create a qsub-like file (in which the job code will be) by copying the qsub header ($scrhead) into the intermediary $QSUBint file

YOUR CUSTOM CODE GOES HERE

Keep track of job names to ensure job dependencies (see below)

Modify the Targets.txt file to mark the job as '_DONE'

Prepare code for the next iteration ($IterateSH). This iteration depends on the completion of all the current jobs ($myJobsVec)

Submit job to cluster and exit
